# Understanding the Crucial Significance of the Temperature and Potential Window on the Stability of Carbon Supported Pt-Alloy Nanoparticles as Oxygen Reduction Reaction Electrocatalysts

**DOI:** 10.1021/acscatal.1c04205

**Published:** 2021-12-13

**Authors:** Tina Đukić, Leonard Jean Moriau, Luka Pavko, Mitja Kostelec, Martin Prokop, Francisco Ruiz-Zepeda, Martin Šala, Goran Dražić, Matija Gatalo, Nejc Hodnik

**Affiliations:** † Department of Materials Chemistry, National Institute of Chemistry, Hajdrihova 19, 1001 Ljubljana, Slovenia; ‡ Faculty of Chemistry and Chemical Technology, University of Ljubljana, Večna pot 113, 1000 Ljubljana, Slovenia; § University of Chemistry and Technology Prague, Technická 5, 166 28 Dejvice, Prague 6, Czech Republic; ∥ Department of Analytical Chemistry, National Institute of Chemistry, Hajdrihova 19, 1001 Ljubljana, Slovenia; ⊥ ReCatalyst d.o.o., Hajdrihova 19, 1001 Ljubljana, Slovenia; # University of Nova Gorica, Vipavska 13, 5000 Nova Gorica, Slovenia

**Keywords:** oxygen reduction reaction (ORR), intermetallic (ordered) platinum alloys, stability, temperature, potential window, redeposition, electrochemical flow cell (EFC), inductively coupled plasma mass spectrometry (ICP-MS)

## Abstract

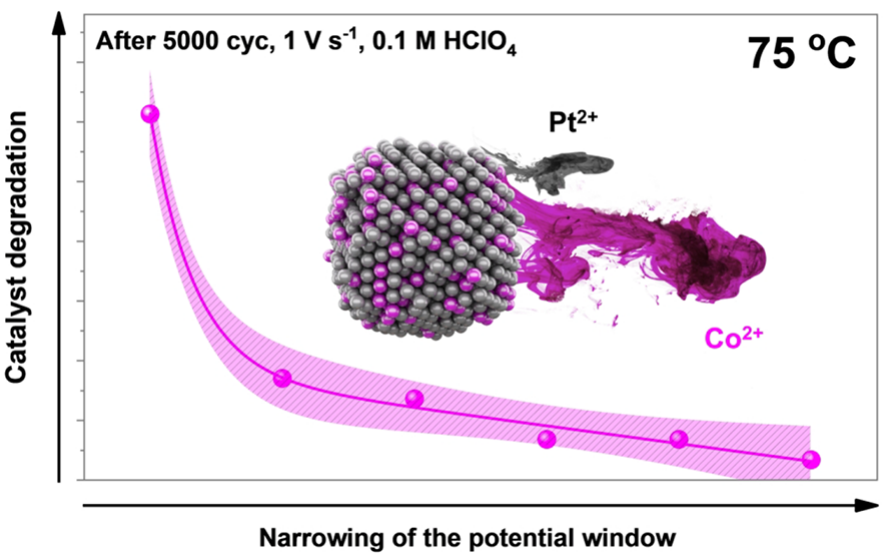

The present research provides a study of carbon-supported intermetallic Pt-alloy electrocatalysts and assesses their stability against metal dissolution in relation to the operating temperature and the potential window using two advanced electrochemical methodologies: (i) the in-house designed high-temperature disk electrode (HT-DE) methodology as well as (ii) a modification of the electrochemical flow cell coupled to an inductively coupled plasma mass spectrometer (EFC-ICP-MS) methodology, allowing for highly sensitive time- and potential-resolved measurements of metal dissolution. While the rate of carbon corrosion follows the Arrhenius law and increases exponentially with temperature, the findings of the present study contradict the generally accepted hypothesis that the kinetics of Pt and subsequently the less noble metal dissolution are supposed to be for the most part unaffected by temperature. On the contrary, clear evidence is presented that in addition to the importance of the voltage/potential window, the temperature is one of the most critical parameters governing the stability of Pt and thus, in the case of Pt-alloy electrocatalysts, also the ability of the nanoparticles (NPs) to retain the less noble metal. Lastly, but also very importantly, results indicate that the rate of Pt redeposition significantly increases with temperature, which has been the main reason why mechanistic interpretation of the temperature-dependent kinetics related to the stability of Pt remained highly speculative until now.

## Introduction

In humanity’s goal to become a carbon-neutral society, mass adoption of hydrogen as the energy carrier and proton exchange membrane fuel cells (PEMFCs) as the energy production technology are becoming recognized as one of the most important pieces of the puzzle in the fight against the negative impacts of climate change.^1−3^ In PEMFCs, hydrogen as a fuel and oxygen from the air are converted into clean electricity with water as the only byproduct. This makes PEMFCs especially suitable for competing with and eventually replacing conventional internal combustion engines (ICEs) in transport related applications. Specifically, while PEMFCs are expected to find their use also in the passenger light-duty vehicles (LDVs), it is becoming increasingly more evident that they can be significantly more competitive for the use in heavier transport-related applications that require longer travel times. Thus, one of the most promising development directions that is starting to receive significant attention is, for instance, the use of PEMFCs in heavy-duty vehicles (HDVs).^4^

However, in comparison to the ICEs, the costs related to the PEMFC technology are still too high. While the cost of most of the parts of the PEMFC will benefit highly from the economies of scale, the costs related to the precious metals, primarily Pt found in the electrocatalyst will not and might even increase at higher PEMFC market penetration.^5^ Most of the Pt is required for enhancing the kinetics of sluggish oxygen reduction reaction (ORR) on the cathode side of the PEMFC. As of today, the only electrocatalyst system to already reach the production phase is comprised of pure Pt nanoparticles (NPs) supported on partly graphitized high-surface area carbons (Pt/C).^6^ However, according to the existing evidence,^7^ mass commercialization of PEMFC technology will not be possible without bringing the Pt amount per vehicle down to the levels comparable with the ICE vehicles. Consequently, significant efforts in the past decades have also gone toward the next electrocatalyst system projected to reach the production phase—the so-called dealloyed Pt-alloys with other, less expensive and less noble 3d transition metals such as Co, Ni or Cu.^6^ Cost reduction using Pt-alloys is possible due to two key features: (i) Pt-alloys dilute Pt-atoms inside the NPs core and thus improve Pt overall utilization^8−11^ and (ii) they promote a higher kinetic activity toward the ORR due to a combination of a ligand, strain, coordination number, and/or surface disorder effects.^12−18^

However, while the activity benefit of Pt-alloys has become rather clear, their commercialization is currently hindered by the lack of understanding of their stability behavior. While electrocatalyst stability is of general importance, its significance becomes even more decisive for their application in HDVs—resulting from in-average longer travel distances in comparison to passenger LDVs and, thus, significantly higher system lifetime requirements.^4^ Because the degradation of Pt-alloy electrocatalysts is caused by various extremely complicated phenomena,^19^ significant efforts have to be invested into clarifying and understanding individual mechanisms. There are two basic groups of degradation mechanisms: (i) electrochemically induced (transient) dissolution of Pt, which is closely related with the dynamics of formation/reduction of the Pt-oxide,^20^ resulting in Ostwald ripening^21^ and/or formation of metallic Pt bands in the membrane;^22^ (ii) electrochemical and chemical carbon support corrosion,^23,24^ leading to the agglomeration and/or detachment of Pt NPs. In the case of Pt-alloy NPs, one also has to deal with the dissolution of the less noble metal.^25−27^ Last but not least, closely connected to the dissolution of Pt and Ostwald ripening, general understanding of Pt redeposition phenomenon in the catalyst layer is also of utmost importance.^28−30^ However, perhaps equally important as improving the intrinsic durability of an electrocatalyst is also understanding how the mentioned degradation mechanisms relate to the different operating conditions in a PEMFC.^31^

For instance, because PEMFCs usually operate at elevated temperatures (60–80 °C^1^), it is highly beneficial to understand the stability behavior of carbon-supported Pt-based electrocatalysts also in terms of the operating temperature. Rare but highly important previous studies focused mainly on the effects of temperature on the kinetics of carbon support corrosion demonstrated that higher rates of carbon oxidation are expected with increasing operating temperature of the PEMFC.^31−33^ However, there also exists a limited number of past studies that address the relation of Pt dissolution and temperature both on Pt NPs^34−38^ as well as bulk Pt.^39−41^ In addition to these studies, Cherevko and co-workers^42^ followed with the first study on the temperature-dependent dissolution of polycrystalline Pt by coupling of the scanning flow cell (SFC) with an inductively coupled plasma mass spectrometer (ICP-MS). SFC-ICP-MS along with other recently used similar methodologies^26,30,43−48^ enables insights into the time-and-potential resolved dissolution of metals, providing yet another dimension to the observed electrochemical signal. Specifically to the above-mentioned work by Cherevko and co-workers,^42^ the authors reported a rather significant influence of temperature on the onsets of both the Pt-oxide formation as well as the reduction. In other words, with an increasing temperature, the onsets of the Pt-oxide formation during the anodic scan on the cyclovoltammogram (CV) shifted to a higher potential, whereas the on-set of the Pt-oxide reduction also shifted toward a higher potentials. However, the differences in the collected time-and-potential resolved Pt dissolution data in relation to the temperature seemed rather insignificant. With increasing temperature, they observed only a slight increase in the anodic dissolution of Pt, while perhaps even more surprisingly they observed even a decrease in the cathodic dissolution of Pt. In other words, in the work by Cherevko and co-workers,^42^ the total amount of dissolved Pt seemed to be almost constant, whereas a prior study by Jerkiewicz and co-workers concluded that the total dissolved amount of Pt with increasing temperature might be even slightly lower.^39^ While this indicates the possibility that Pt might be stabilized with increasing temperature, Cherevko and co-workers also stated the mechanistic interpretation of the temperature-dependent kinetics remains highly speculative.^42^ In addition to that, they have provided the idea that perhaps the decrease in the cathodic dissolution of Pt with higher temperature could be a result of an increased rate of Pt redeposition.^28−30^ With no follow-up studies further addressing the mechanistic interpretation of temperature dependence of Pt dissolution, many open questions remained. For instance, does the rate of Pt redeposition increase or decrease with an increasing operating temperature? Also, how does the relation between Pt dissolution and operating temperature impact the dissolution of the less noble metal in the case of Pt-alloys? In addition to these questions, another potentially unresolved conception in the fuel cell community is also whether the growth of the Pt NPs observed at the elevated temperatures is due to carbon support corrosion and successive Pt agglomeration or due to Pt Ostwald ripening?^49^ Most reports explain their results by the logical notion that corrosion of carbon support becomes massive at temperatures approaching real fuel cell conditions.^50,51^ Furthermore, other studies,^32^ including one of our recent studies making use of a physical model,^52^ once again also suggest Pt redeposition. While it is far from trivial to answer all of these questions, additional complexity can be introduced when one considers also the importance of the operating voltage/potential. There are a few (but very important) examples in the literature that provide evidence that both the upper voltage/potential limit (UVL/UPL), as well as the lower voltage/potential limit (LVL/LPL) are important for limiting the degradation of Pt-based carbon supported electrocatalysts in the PEMFC. For example, Uchimura and co-workers^53^ provided evidence of increasing electrochemically active surface area (ECSA) losses upon performing 15 000 accelerated degradation test (ADT) cycles under H_2_–N_2_ feed (Anode/Cathode, respectively) at 80 °C and 100% RH in 25 cm^2^ fuel cells using a fixed UVL of 0.95 V while gradually lowering the LVL from 0.8 V toward the 0.6 V. The authors have attributed this to increased Pt dissolution resulting from anodic Pt-oxide formation followed by only some cathodic Pt-dissolution due to destabilization of the previously formed oxide species. Years later, the transient nature of Pt-dissolution was experimentally demonstrated by Topalov and co-workers with SFC-ICP-MS.^54^ As a part of the Toyota Mirai launch in 2015, Yoshida and co-workers^55^ affirmed the necessity of limiting both the UVL and the LVL in order to minimize the ECSA losses during fuel cell operation. Further revelations followed with the Department of Energy (DoE) Mirai Fuel Cell Vehicle report that has shown system-level limitations on the LVL of the Mirai fuel cell stack.^56^ In addition, Todoroki and co-workers provided important evidence in their model core–shell system comprising 4 monolayers of Pt deposited on the Pd(111) surface. They have shown that lowering of the UPL from 1.0 V to 0.85 or 0.8 V resulted in significant retention of the activity for ORR.^57^ Last but not least, while avoiding high UVLs as a consequence of start-up/shut-down conditions is important mostly for avoiding severe carbon corrosion,^58^ our recent work that we will soon publish in a separate publication shows that especially for Pt-alloy cathodes, the choice of LVL/LPL plays a decisive role in extending the PEMFC lifetime. Namely, a profound difference in voltage degradation at 1.5 A cm^–2^ in a 50 cm^2^ single-cell has been observed during only a thousand ADT cycles (1000 cycles, 0.925–0.X V_RHE_; X = 70/60/50, 3 s hold at both LVL and UVL; ambient outlet pressures, stoichiometry 1.5/2, dew point 50 °C anode and cathode; H_2_/N_2_) in the case where the LVL was lowered from 0.7 to 0.6 or even 0.5 V. Interestingly, for all three LVL, the rate of voltage degradation increased with increasing operating temperature during the ADT. In addition, as part of the same work, the degradation effect has also been observed with the electrochemical flow cell coupled (EFC) to an ICP-MS. What we have noticed is that with decreasing LPL (from 0.7 to 0.65 to 0.6 V), an increase in cathodic dissolution of Pt occurs. This was directly followed by an increase in the cathodic dissolution of the less noble metal. Thus, we have concluded that the dissolution of the less noble metal is closely connected with the dynamics of the Pt-oxide formation and reduction. UVL/UPL corresponds to the amount of anodically formed Pt-oxide, which is then followed by now already well-known oxide-place exchange mechanism^54,59^ that results in cathodic dissolution of Pt. In the case of Pt-alloys, cathodic and anodic dissolution of Pt is then always followed by also dissolution of the less noble metal.^25,26^ The rate of this process is then defined by the UVL/UPL—the lower we go, the more Pt-oxide we reduce, subsequently triggering a higher degree of metal dissolution. While this might not be of a particular importance for pure Pt cathodes, aging of the Pt-alloy cathode will result in not only the decrease in kinetic performance but also additional degradation phenomenon related with the presence of the less noble metal ions in the catalyst layer and/or the membrane.^60,61^

**Scheme 1:**
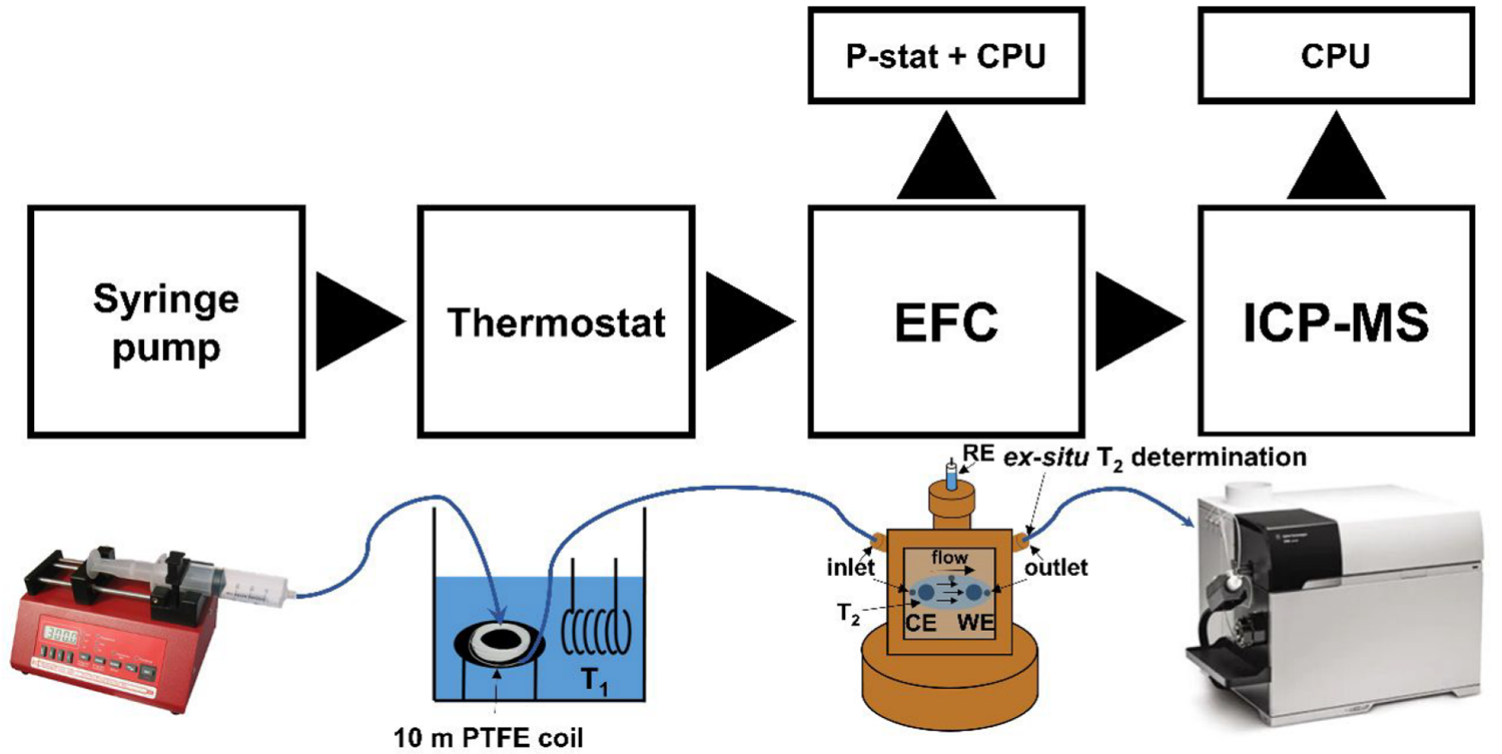
HT-EFC-ICP-MS Setup Used for Obtaining Time-and-Potential Resolved Metal Dissolution at Various Temperature and Other ADT Conditions

The present research provides a study of carbon-supported intermetallic Pt-alloy electrocatalysts and assesses their stability against metal dissolution in relation to the operating temperature and the potential window using two advanced electrochemical methodologies. For the stability assessment, proprietary intermetallic Pt–M catalysts from ReCatalyst d.o.o. were developed on the basis of the work published elsewhere.^33^ In the first part, a study is conducted using our previously reported and in-house designed high-temperature disc electrode setup (HT-DE).^31^ The HT-DE setup enables one to perform ADTs at various elevated temperatures by using a reflux cooling condenser in order to avoid evaporation of the electrolyte (in our case 0.1 M HClO_4_). Furthermore, specific activity (SA), mass activity (MA), and electrochemically active surface area normalized via CO-electrooxidation (ECSA_CO_) before and after the ADT are evaluated using a typical thin-film rotating disc electrode setup (TF-RDE). In addition to the electrochemical evaluation of the electrocatalyst, one can also determine the amount of dissolved less noble metal by sampling the electrolyte after the ADT using ICP-MS. In order to further complement the findings of the HT-DE study and obtain mechanistic insights, an already well-established^26,30,43,44,46^ highly sensitive (ppb range) electrochemical flow cell coupled to an inductively coupled plasma mass spectrometer (EFC-ICP-MS) methodology is used in the second part of this work to enable time-and-potential resolved measurements of metal dissolution. The latter has been for the purpose of the study additionally upgraded to enable investigation of metal dissolution at various temperatures (Scheme 1; hereinafter referred to as HT-EFC-ICP-MS).

## Experimental Section

### Synthesis of the Intermetallic d-int-Pt-M/C Electrocatalysts

The ReCatalyst electrocatalysts were prepared in accordance with the processes already reported previously.^33,62,63^ Briefly, the electrocatalysts have been prepared in three steps. In the first step, Pt NPs were deposited onto a commercial carbon black support (Ketjen Black EC300J) via double passivation galvanic displacement method reported elsewhere.^33^ In the second step, the prepared composites with deposited Pt NPs were thermally annealed in order to obtain an intermetallic crystal phase. In the last step dealloying (acid washing) was performed in accordance to the work described previously.^64−66^

### XRD Analysis

The powder X-ray diffraction (XRD) measurements of samples containing Co were carried out on a PANalytical X’Pert PRO diffractometer with Cu Kα radiation (λ = 1.541874 Å) in the 2θ range from 10° to 60° with the 0.039° step per 300 s using a fully opened Pixcel detector.

The powder X-ray diffraction (XRD) measurements of samples containing Ni and Cu were carried out on a PANalytical X’Pert PRO MPD diffractometer with Cu Kα1 radiation (λ = 1.5406 Å) in the 2θ range from 10° to 60° with the 0.034° step per 100 s using a fully opened X’Celerator detector. Samples were prepared on a zero-background Si holder.

### Transmission Electron Microscopy (TEM) Analysis

STEM imaging was carried out in a probe Cs-corrected scanning transmission electron microscope Jeol ARM 200 CF operated at 80 kV.

### ICP-OES and Digestion Procedure for Metal Loading Determination in the Electrocatalyst Powders

All reagents used were of analytical grade or better. For sample dilution and preparation of standards, ultrapure water (18.2 MΩ cm^–1^, Milli-Q, Millipore) and ultrapure acids (HNO_3_ and HCl, Merck-Suprapur) were used. Standards were prepared in-house by dilution of certified, traceable, inductively coupled plasma (ICP)-grade single-element standards (Merck CertiPUR). A Varian 715-ES ICP optical emission spectrometer was used. Prior to ICP-OES analysis, each electrocatalyst was weighted (approximately 10 mg) and digested using a microwave-assisted digestion system (Milestone, Ethos 1) in a solution of 6 mL HCl (conc.) and 2 mL HNO_3_ (conc.). Samples were then filtered, and the filter paper was again submitted to the same digestion protocol. These two times digested samples were cooled to RT and then diluted with 2%v/v HNO_3_ until the concentration was within the desired concentration range.

### Accelerated Degradation Tests Using the High-Temperature Disk Electrode Methodology (HT-DE)

#### High-Temperature Disk Electrode (HT-DE) Setup

The accelerated degradation tests (ADTs) were performed in a setup already described as a part of our previous work (see also SI, Figure S1).^31^ Briefly, the setup is composed of a two-compartment HT-cell using 0.1 M HClO_4_ electrolyte (Carl Roth, Rotipuran Supra) with a conventional three-electrode system controlled by a potentiostat (CompactStat, Ivium Technologies). The reversible hydrogen electrode (HydroFlex, Gaskatel) was used as a reference (separated from the working electrode in a different compartment via a salt-bridge), and a graphite rod was used as a counter electrode (with respect to the temperature at which ADTs were performed, a fresh graphite rod was used for each measurement).

#### Thin-Film Rotating Disk Electrode (TF-RDE) Setup

While the ADTs were performed in the HT-DE setup, oxygen reduction reaction (ORR) polarization curves and CO-electrooxidation CVs both before as well as after the ADT were measured in a typical TF-RDE setup also in accordance to our previous work.^31^ The electrochemical measurements were conducted with a CompactStat (Ivium Technologies) in a two-compartment electrochemical cell in a 0.1 M HClO_4_ electrolyte with a conventional three-electrode system. Similarly, a reversible hydrogen electrode (HydroFlex, Gaskatel) was used as a reference, and a graphite rod was used as a counter electrode (again, with respect to the temperature at which ADTs were performed, a fresh graphite rod was used for each measurement).

#### Preparation of the Thin Films and the Setups

The extensive cleaning was performed in order to eliminate any organic and inorganic impurity contributions that could potentially affect the stability of the studied electrocatalyts. Prior to the set of degradation experiments, all the glassware was soaked in both a base bath (mixture of KOH and isopropanol) and an acid bath (mixture of conc. HNO_3_ and H_2_SO_4_) as well as boiled in distilled water 3 times. Prior to each experiment, the HT-cell was heated for 2 h at 90 °C in 0.1 M HClO_4_ and then boiled in Milli-Q water for 2 h, whereas the RT-cell was boiled in distilled water for 1 h.

The working electrode was a glassy carbon (GC) disk embedded in Teflon (Pine Instruments) with a geometric surface area of 0.196 cm^2^. The GC electrode was polished to a mirror finish with Al_2_O_3_ paste (particle size 0.05 μm, Buehler) on a polishing cloth (Buehler). After it was polished, the electrode was rinsed and ultrasonicated (Ultrasound bath Iskra Sonis 4) in Milli-Q/isopropanol mixture several times for 5 min. Once the GC electrode is prepared, 20 μL of 1 mg mL^–1^ fresh prepared water-based well-dispersed electrocatalyst ink was pipetted on the electrode to completely cover it, and it was dried under ambient conditions. After the drop had dried, 5 μL of Nafion solution (ElectroChem, 5% aqueous solution) diluted in isopropanol (1:50) was added. The electrode was then mounted on the rotator (Pine Instruments).

#### Electrochemical Characterization

The electrode was then initially placed in the TF-RDE setup in an inert gas saturated electrolyte (0.1 M HClO_4_) under potential control at 0.05 V_RHE_ using a rotator (Pine technologies). All electrocatalysts were then electrochemically activated (50 cycles between 0.05 and 1.2 V_RHE_ with a scan rate of 300 mV s^–1^ under a rotation rate of 600 rpm). After the activation, the electrolyte was exchanged for a fresh one. ORR polarization curves were measured in an oxygen saturated electrolyte with rotation at 1600 rpm in the potential window 0.05–1.0 V_RHE_ with a scan rate of 20 mV s^–1^. At the end of ORR polarization curve measurement, the electrolyte was purged with CO under potentiostatic mode (0.05 V_RHE_) in order to ensure successful CO adsorption. Afterward, the remaining CO in the electrolyte had been displaced, and the electrolyte was saturated with Ar. CO-electrooxidation was performed using the same potential window and scan rate as in ORR but without rotation and in an Ar-saturated electrolyte. Electrochemical surface area (ECSA_CO_) was determined by integrating the charge in CO-electrooxidation (“stripping”) experiments as described in ref (67). For ORR, after subtraction of the background current (due to capacitive currents), kinetic parameters were calculated at 0.9 V_RHE_. Ohmic resistance of the electrolyte was determined and compensated for as reported in ref (68). Afterward, the working electrode was carefully transferred to the HT-DE setup (taking care to not introduce any impurities during the transfer process), and an ADT was performed, which comprised 5000 cycles at various temperatures (RT, 50 and 75 °C) and potential windows (X-Y V_RHE_; X = 0.4, 0.6 and 0.7; Y = 1.2, 1.0, 0.925; 5000 cycles, 1 V s^–1^, 0.1 M HClO_4_). Afterward, the working electrode was again carefully transferred back to the standard TF-RDE setup, and the ORR polarization curve as well as CO-electrooxidation were measured once again (again at RT). In addition, after each ADT, the electrolyte from the HT-cell was sampled (in 15 mL vial) for ex situ determination of Co using ICP-MS.

#### Ex Situ ICP-MS for the Determination of Metals in the Electrolyte after ADTs

Ex situ samples for determination of metal concentrations were collected after the ADTs and analyzed using mass spectrometry with inductively coupled plasma. Samples were not diluted prior to measurement and were measured as received. For the preparation of standards, ultrapure water (Milli-Q, Millipore) and ultrapure acid (HClO_4_; Carl Roth, Rotipuran Supra) were used. Standards were prepared in-house by dilution of certified, traceable, inductively coupled plasma (ICP) grade single-element standards (Merck Certipur). An Agilent quadrupole ICP-MS instrument (Agilent 7900, Agilent Technologies, Santa Clara, CA) equipped with a MicroMist glass concentric nebulizer and a Peltier-cooled, Scott-type spray chamber was used for the measurements. Each ex situ electrolyte sample was measured three times, and the RSD for each measurement was determined. A typical RSD for Co was 3%, whereas the amount of dissolved Pt was too low for accurate and relevant ex situ determination.

### High-Temperature Electrochemical Flow Cell Coupled to Inductively Coupled Plasma Mass Spectrometry (HT-EFC-ICP-MS)

#### Electrochemical Flow Cell (EFC)

The setup and measurement guidelines were established as part of the previous work.^25,26,29,30,43,44,69,70^ Briefly, the working and counter electrodes in the electrochemical flow cell (EFC) were glassy carbon discs (3 mm diameter) embedded into PEEK material (BASi). The discs were aligned in series; the counter electrode was placed first and the working electrode second in the direction of the electrolyte flow. The sample was deposited on the electrode by drop casting a 5 μL drop of the ultrasonically homogenized catalyst ink (1 mg mL^–1^). Such preparation resulted in the electrocatalyst loading of 5 μg for all electrocatalysts. In addition, in order to increase the surface area of the counter electrode, a 5 μL drop of Ketjen Black EC300J suspension (1 mg mL^–1^) was deposited on the glassy carbon counter electrode. After the drop had dried, 5 μL of Nafion solution (ElectroChem, 5% aqueous solution) diluted in isopropanol (1:50) was added, covering both electrodes at the same time. The Ag|AgCl reference electrode potential against RHE was determined before the start of the experiment. The housing of the cell was made from PEEK material, and the design was modeled after a commercial cross-flow cell (BASi, MF-1092, cross-flow cell). The volume of the cell was established with a homemade silicon gasket with 1.0 mm thickness and 1.5 cm^2^ ellipsoidal cut. The carrier solution (0.1 M HClO_4_, degassed) was first pumped through a 10 m long PTFE tube (1/16″ OD × 1.0 mm ID, BGB Analytik Vertrieb GmbH) immersed in temperature-controlled (MGW Lauda thermostat) distilled water before going through the cell (Scheme 1; see also SI, Figure S2). This enabled the electrolyte (0.1 M HClO_4_) sufficient time to reach the desired temperature for each experiment. Lastly, after each measurement, the actual temperature of the electrolyte was determined at the outlet of the EFC using a thermocouple (TJC1-CAXL-IM025U-150-SMP-M, Omega), whereas the path of the tubing upon exiting the water in the thermostat and the inlet of the EFC was kept constant. For the RT measurement, the thermostat was turned off. Additionally, in order to avoid possible memory effects, each temperature (RT, 50 °C, 75 °C) and each measurement protocol (lower potential limit; LPL or wide potential window; WPW) were performed on a fresh catalyst film. For each temperature (RT, 50 °C, 75 °C) and potential window (LPL or WPW), each experiment was performed at least two times for reproducibility. The flow was kept at a constant at 400 μL min^–1^ for all experiments using a WPI AL1000 syringe pump. The Ag|AgCl reference correction to RHE was adjusted on the basis of the measured temperature accordingly to ref (42).

#### ICP-MS

The EFC was coupled with an ICP-MS detector, namely an Agilent 7900ce ICP-MS instrument (Agilent Technologies, Palo Alto, CA) equipped with a MicroMist glass concentric nebulizer and a Peltier cooled Scott-type double-pass quartz spray chamber. The signals were recorded for Cu^63^, Ni^60^, Co^59^, and Pt^195^ with 0.5 s integration per data point. To convert the ICP-MS signals to concentration (ppb), a standard solution of Cu, Ni, Co, and Pt in 0.1 M HClO_4_ were recorded with the following concentrations: 0.5, 1, 2, 5, 10, 20, 50, and 100 ppb.

#### Electrochemical Protocol

Electrochemical experiments were performed with a CompactStat (Ivium Technologies) with a typical three-electrode setup. No ohmic drop compensation method was used. Initially, Milli-Q water was pumped through the cell under open circuit conditions (OCP) before switching to 0.1 M HClO_4_. After a steady background has been reached (for at least 2 min), the potentiodynamic protocol was started; in order to check for the effect of the lower potential limit, the electrocatalysts were cycled for three cycles between 0.925 and *X* V_RHE_ (*X* = 0.7, 0.65, and 0.6) with nine cycles in total (scan rate of 5 mV s^–1^). In both cases, the experiment was followed with two cycles between 0.05 and 1.4 V_RHE_ (scan rate of 5 mV s^–1^). After each experiment, a sequence of potential pulses was performed in order to synchronize the electrochemical experiment with the ICP-MS signal.

## Results and Discussion

**Figure 1:**
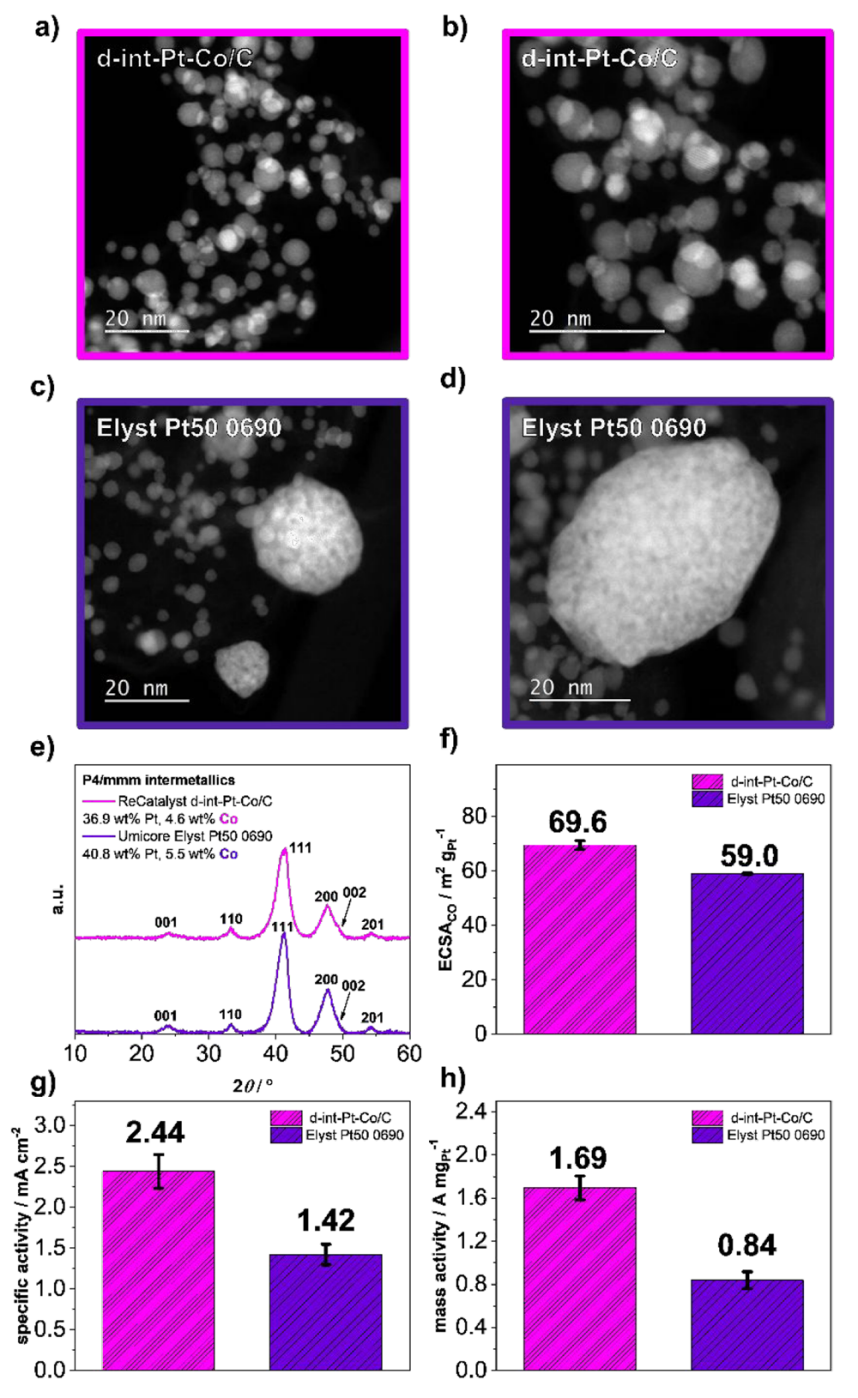
(a–d) HAADF TEM, (e) XRD, and (f–h) TF-RDE comparison between ReCatalyst d-int–Pt-Co/C electrocatalyst and Umicore Elyst Pt50 0690 Pt-Co/C benchmark. Additional characterization is available in the SI, Figures S3–S5. In all figures, magenta is used for the data corresponding to the experimental ReCatalyst electrocatalyst, whereas the data corresponding to the Umicore benchmark is in purple.

Figure 1 provides a comparison between the experimental d-int-Pt-Co/C electrocatalyst from the ReCatalyst and a commercial Pt–Co benchmark from Umicore (Elyst Pt50 0690). High-annular dark-field TEM imaging from Figure 1a–d (see also SI, Figures S3–S4) provides a general idea of the particle sizes. However, the overlap in the XRD spectra (Figure 1e) provides evidence that both the bulk chemical composition (Pt:Co ratio), as well as the crystal structure (intermetallic tetragonal *P*4/*mmm* crystal structure) of both the ReCatalyst experimental electrocatalyst and the Pt–Co benchmark from Umicore are similar. The similarity of the Pt:Co chemical composition was additionally confirmed by the ICP-OES results obtained from the digestion of both electrocatalysts. In addition, while the information is not readily available directly by Umicore, our TEM analysis (see SI, Figure S4) along with the stability data presented later as part of this work provides strong indications that both electrocatalysts also most likely use the same carbon support (Ketjen Black EC300J). Figure 1f–h provide a comparison of the initial TF-RDE performance (see also SI, Figure S5), where the ReCatalyst electrocatalyst exhibits both a higher ECSA_CO_ and a higher SA than the Umicore benchmark, as well as subsequently higher MA. We presume that the difference in the ECSA_CO_ can most likely be attributed to the different methods of the deposition of Pt NPs used by Umicore in contrast to ReCatalyst—the double passivation with galvanic displacement method reported previously.^33^ In contrast to the typically used methods, the double passivation method allows for intrinsically better dispersion by allowing for crystallization of the Pt NPs directly on the carbon support, thus combining the usually sequential nature of Pt NPs synthesis and deposition steps into a single step. Thus, on the basis of the TEM imaging from Figure 1a–d (see also SI, Figures S3–S4), one could attribute this difference to the absence of the oversized (>20 nm) Pt–Co NPs in the case of the ReCatalyst electrocatalyst. However, the benefit in terms of the SA, while currently unclear, could perhaps be attributed to slight differences in the thermal annealing and/or dealloying steps.

Prior to the interpretation of the results presented as part of the stability study, it is important to state that the main goal of this work was to investigate the stability of the ReCatalyst intermetallic Pt–Co/C electrocatalyst while obtaining the understanding related to the effects of both the temperature and the potential window. However, in the last part of the assessment, the ReCatalyst electrocatalyst was additionally compared with the Umicore benchmark in order to validate that the stability of the ReCatalyst electrocatalyst is indeed corresponding to the current state-of-the-art.

**Figure 2:**
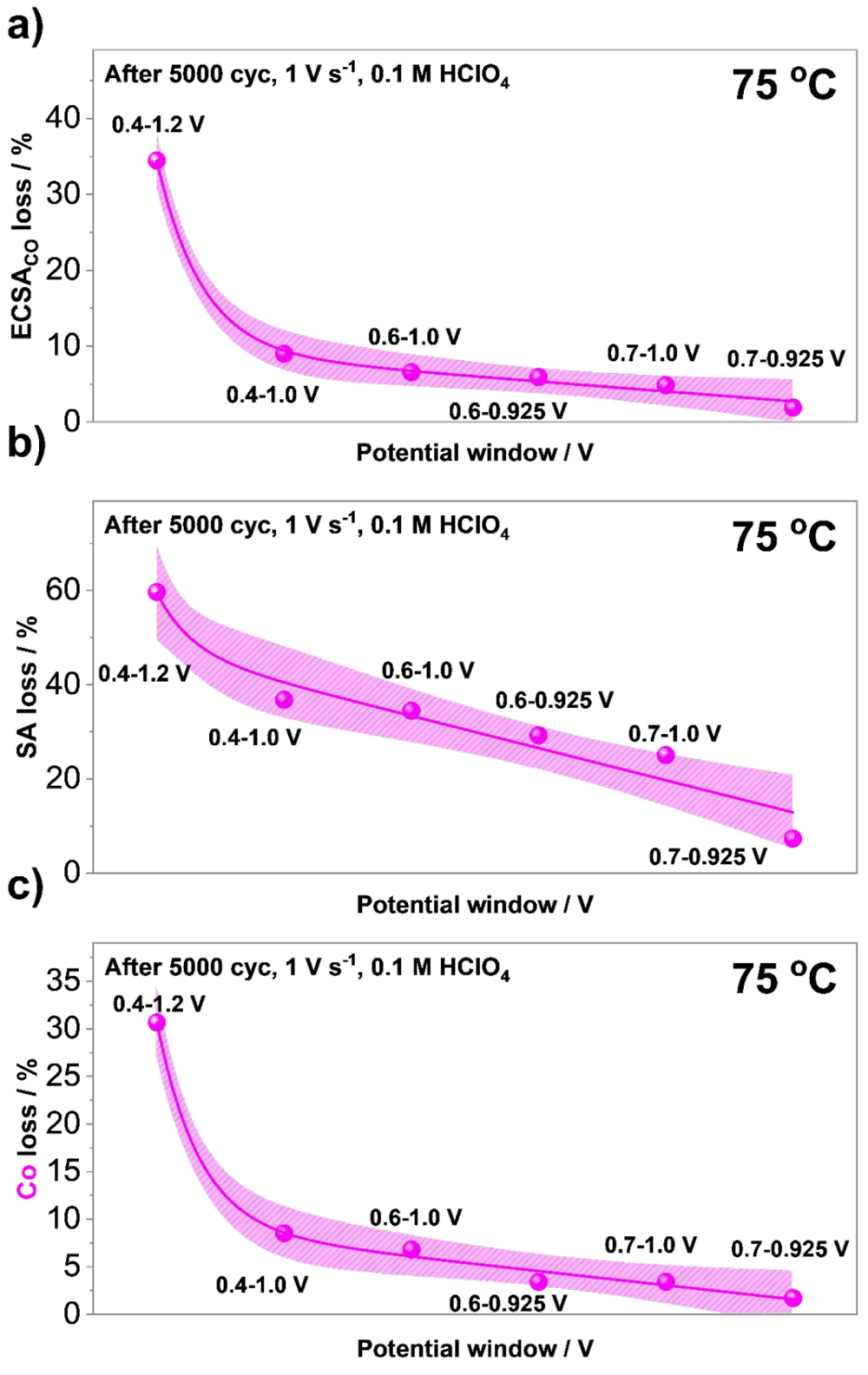
Effect of varying the potential window on the accelerated degradation tests (ADTs) performed at a constant temperature (75 °C, 5000 cycles, 1 V s^–1^, 0.1 M HClO_4_) comparing (a) ECSA_CO_ loss, (b) SA loss, and (c) Co loss of the ReCatalyst intermetallic dealloyed Pt–Co/C electrocatalyst. Electrochemical data can be found in SI, Figures S6–S7. The experimental data in this figure was fitted only for the purpose of better visualization.

Figure 2 shows the assessment using the impact of the potential window (*X*-*Y* V_RHE_; *X* = 0.4, 0.6, and 0.7; *Y* = 1.2, 1.0, and 0.925; 5000 cycles, 1 V s^–1^, 0.1 M HClO_4_) at a constant (elevated) temperature of 75 °C. The results are rather self-explanatory and in line with the previous studies conducted at RT.^46,71^ However, while the prior studies provide evidence in relation to the dissolution of Pt and Co in relation to the potential window, the present study using the HT-DE methodology provides information closer to the real operational conditions by performing the ADTs at 75 °C. The results indicate that narrowing of the potential window results in a lower loss of ECSA_CO_, SA as well as Co. Not only that, but in the case of ECSA_CO_ (Figure 2a) and loss of Co (Figure 2b), a clear linear relationship is observed in the case of all measured potential windows with the exception of the harshest ADT between 0.4 and 1.2 V_RHE_. This is in line with the previous observations related to the transient dissolution of Pt, where in contrast to UPLs of 1.0 V or lower, an order of magnitude difference has been observed when increasing the UPL to 1.2 V_RHE_ or higher.^20,25,26,30^ While cathodic dissolution of Pt as a consequence of the place-exchange mechanism between Pt and O^54^ seems to already dominate at relatively low UPLs (as low as 0.925 V_RHE_),^46^ the results presented in Figure 2 suggest that in the case of Pt-alloys, any leaps such as the ones experienced during the start-up/shut-down conditions^71^ should be avoided at all cost. In addition, a significant difference is also experienced when the UPL is further reduced to 0.925 V_RHE_ and the LPL to 0.7 V_RHE_. While the mechanistic details related to the LPL effect will be reported in a separate publication in the near future, the present results already clearly indicate a clear relation such that lowering of not only the UPL but also the LPL limits the degradation of Pt-alloy electrocatalysts. The main challenge that arises at this point is, however, how to avoid sacrificing a big part of the maximum power density in a single-cell setting as well as close to realistic operational conditions (air, lower humidity, etc.) when the LPL is limited to 0.7 rather than 0.6 V? One way on how to partly solve this is by using higher Pt loadings at the cathode. This will, in contrast to passenger LDVs (with expected cathode loadings of 0.1 mg_Pt_ cm^–2^ or lower), work better for the application of Pt-alloys in HDVs (with expected cathode loadings of 0.25 mg_Pt_ cm^–2^).^4^ Furthermore, heavier transport applications, in general, could significantly benefit from limiting both the upper and lower voltage limits^55^ and significantly increase the life as well as the long-term performance of the PEMFC stack.

**Figure 3:**
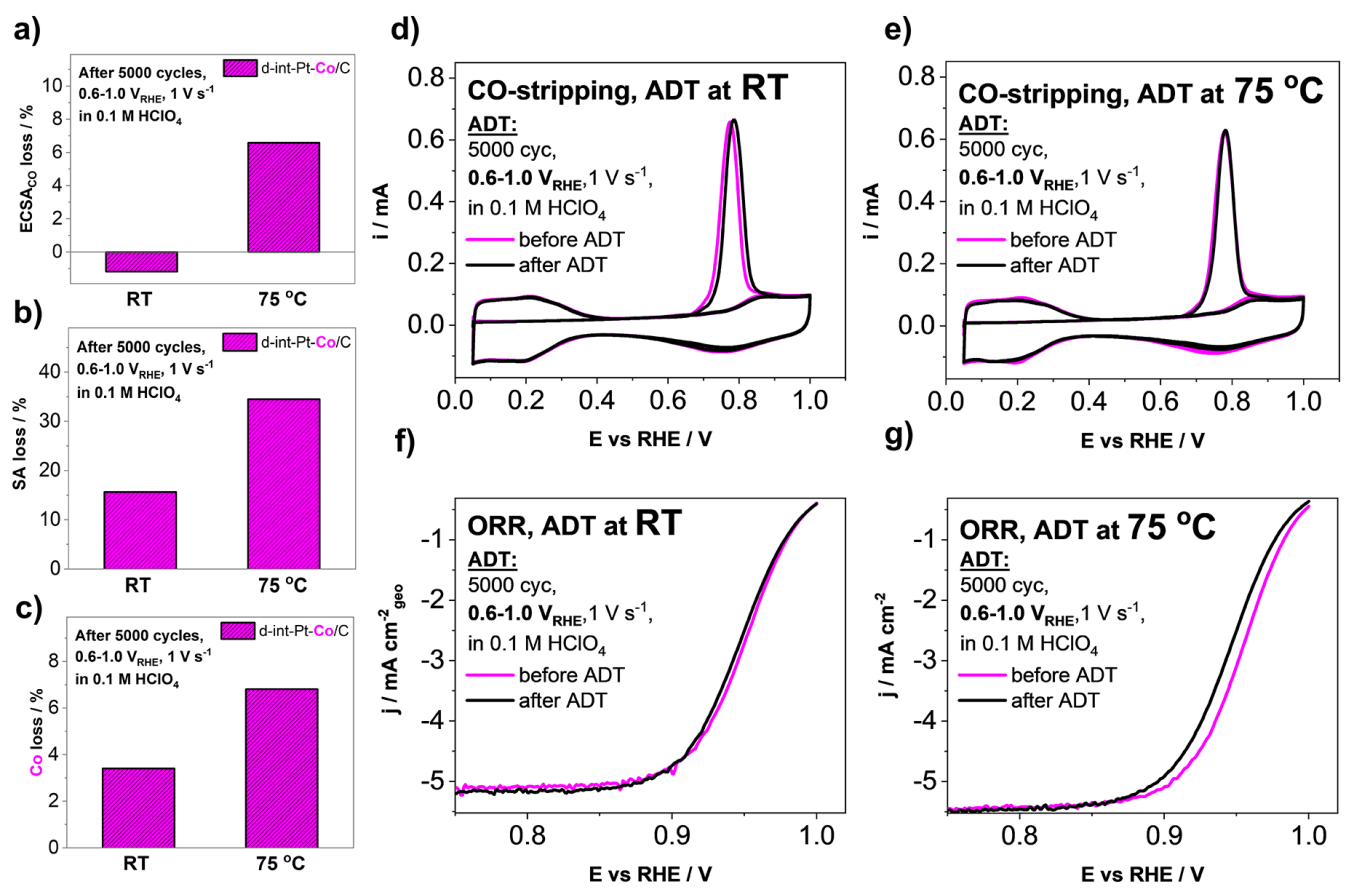
Effect of the temperature on the accelerated degradation tests (ADTs) performed at the potential window of 0.6–1.0 V_RHE_ (comparing RT and 75 °C, 5000 cycles, 1 V s^–1^, 0.1 M HClO_4_) comparing (a) ECSA_CO_ loss, (b) SA loss, and (c) Co loss of the ReCatalyst intermetallic dealloyed Pt–Co/C electrocatalyst. Comparison of the CO-electrooxidation CVs as well as the follow-up cycles before and after the ADT performed at (d) RT and at (e) 75 °C. Comparison of the ORR polarization curves before and after the ADT performed at (f) RT and at (g) 75 °C.

Following the assessment of various potential windows at a constant temperature of 75 °C in Figure 2, we are now focusing only on the potential window that is still most typically used in the literature (several thousands of cycles between 0.6 and 1 V_RHE_) and often referred to as the “operating conditions”.^9,72−75^ In order to demonstrate the crucial importance of the temperature already in this potential range, we are providing a comparison between the measurement performed at the RT and the measurement performed at 75 °C (Figure 3). Figure 3a–c provide evidence that already in this relatively narrow potential window, the temperature significantly impacts the assessment of the electrocatalyst in the case of not only ECSA_CO_ but also the SA as well as loss of Co. In addition, Figure 3d,e provide a comparison of CO-electrooxidation CVs as well as the follow-up cycles before and after the ADT, whereas Figure 3f,g compare the ORR polarization curves in the same manner. Comparison of the CO-electrooxidation shows that the overlap of the CVs before and after the ADT is much closer at RT than at 75 °C. This is in line with the comparison of the ECSA_CO_ (Figure 3a), where in fact the retained ECSA_CO_ after the ADT slightly increases, whereas in the case of 75 °C, the ECSA_CO_ clearly dropped. The increase in ECSA_CO_ resulting from the ADT performed at RT can be explained that the degradation mechanisms contributing to the loss of ECSA_CO_ (e.g., Ostwald ripening) most likely had a lower contribution as the mechanisms that can potentially even increase it (e.g., formation of pores). In the case of the comparison of ORR polarization curves before and after the ADT, we notice that at RT (Figure 3f), the polarization curves remain nicely overlapped with almost no difference observed also in the overpotential region. Furthermore, when the same ADT is performed at 75 °C (Figure 3g), an increase in overpotential is observed after the ADT, while the slopes of both polarization curves before and after the ADT remained similar (indicating no significant contributions from impurities). Thus, similarly to the electrocatalyst durability assessment guidelines provided as part of our previous work,^31^ the stability evaluation of Pt-alloy electrocatalysts at the potential window of 0.6–1 V_RHE_ and RT conditions when using TF-RDE setups is less corresponding to the fuel cell operation and should be performed in half-cell setups at elevated temperatures for the correct assessment of novel electrocatalyst stability. Lastly, the higher drop in SA at 75 °C in contrast to RT (Figure 3b,f,g) is a consequence of an increased loss of Co (Figure 3c), which is in line with the loss of the beneficial ORR enhancement effects induced by Co to the Pt surface.^27,76−78^

**Figure 4:**
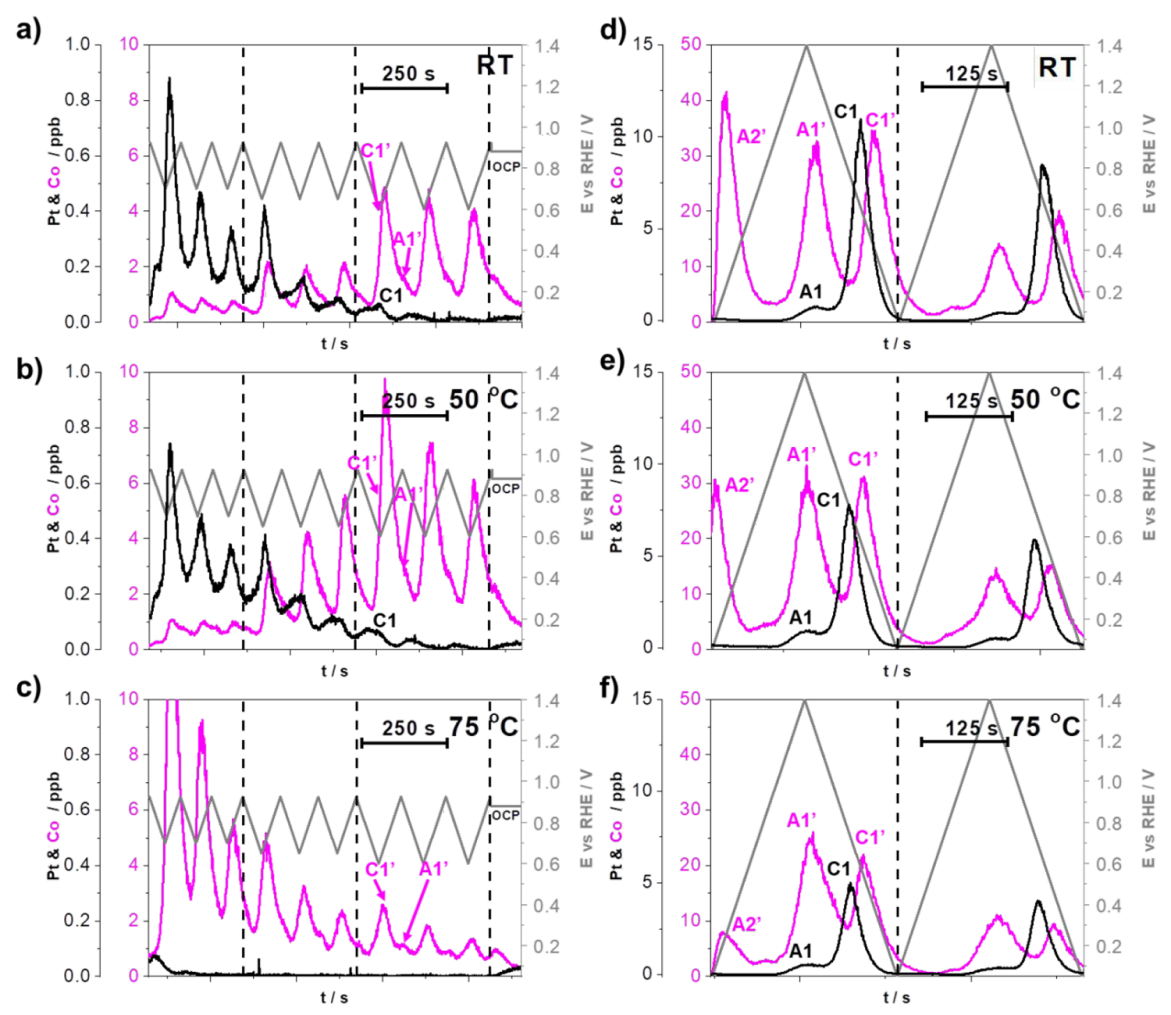
(a–c) Effect of temperature (RT, 50 °C, 75 °C) on the metal dissolution (Pt and Co) during the LPL cycles (0.925-*X* V_RHE_; *X* = 0.7, 0.65, and 0.6; 5 mV s^–1^) and (d–f) WPW cycles (two cycles between 0.05 and 1.4 V_RHE_, 10 mV s^–1^) demonstrated using the HT-EFC-ICP-MS setup in the flow of 0.1 M HClO_4_. Each metal has its own *Y* axis to better compare the profiles despite the detected concentration differences. A1, A1′, A2, and A2′ represent peaks corresponding to anodic dissolution, whereas C1, C1′, C2, and C2′ represent peaks corresponding to the cathodic dissolution of Pt and Co, respectively. The gray zigzag line represents the cycles from LPL to UPL. Transition between different series of cycles is denoted by the dashed lines. Demonstration of the same effect on the experimental intermetallic dealloyed Pt–Cu/C and Pt–Ni/C electrocatalysts is available in the SI, Figures S8–S9, whereas the methodology reproducibility example is provided in SI, Figure S10. Supplementary experiment at RT of fixing the LPL to 0.6 V_RHE_ and increasing the UPL from 0.925 to 1.0 V_RHE_ every three cycles instead is presented in the SI, Figure S11.

Before the evaluation of the results presented in Figure 4, it is important to clarify clear distinctions between the prior temperature-dependent study performed by Cherevko and co-workers:^42^ (i) The prior study was performed on polycrystalline-Pt; (ii) Due to the low specific surface area of poly-Pt, all the measurements have been performed in a very wide potential window (between 0.5 and 1.6 or even 1.9 V_RHE_). This is because both the low specific surface area and the “bulk-like” behavior of the poly-Pt provide for a significantly lower signal for Pt dissolution in contrast to what can be expected in the case of Pt-based nanoparticles.;^20^ (iii) As shown in one of the recent publications by Ehelebe and co-workers,^28^ the thickness of the catalyst layer has a significant contribution to the redeposition of Pt. In other words, the thicker the catalyst layer, the longer the travel path for the dissolved Pt ions and the higher the probability for Pt redeposition. Thus, in this work, it can be expected that any Pt redeposition effects are most likely much more pronounced when dealing with electrocatalyst films composed of composites between carbon and Pt-based NPs. Perhaps even more importantly, there is a significant benefit of not only measuring NPs rather than polycrystalline disks but also measuring Pt-alloy NPs rather than pure Pt NPs. While more will be explained in continuation, the main reasoning behind this is that while Pt is well-known to experience significant redeposition,^28−30^ the less noble metals with a significantly lower standard redox potential such as Co or Ni do not.^77^ Furthermore, in accordance with the evidence provided in our previous publications,^25,26^ anodic and cathodic dissolution of Pt are always followed by anodic and cathodic dissolution of the less noble metal.^25,26^ In other words, regardless of any Pt redeposition effects, being able to measure the dissolution of the less noble metal can be taken as an indication or a probe for what is happening with the dissolution of Pt.

**Scheme 2:**
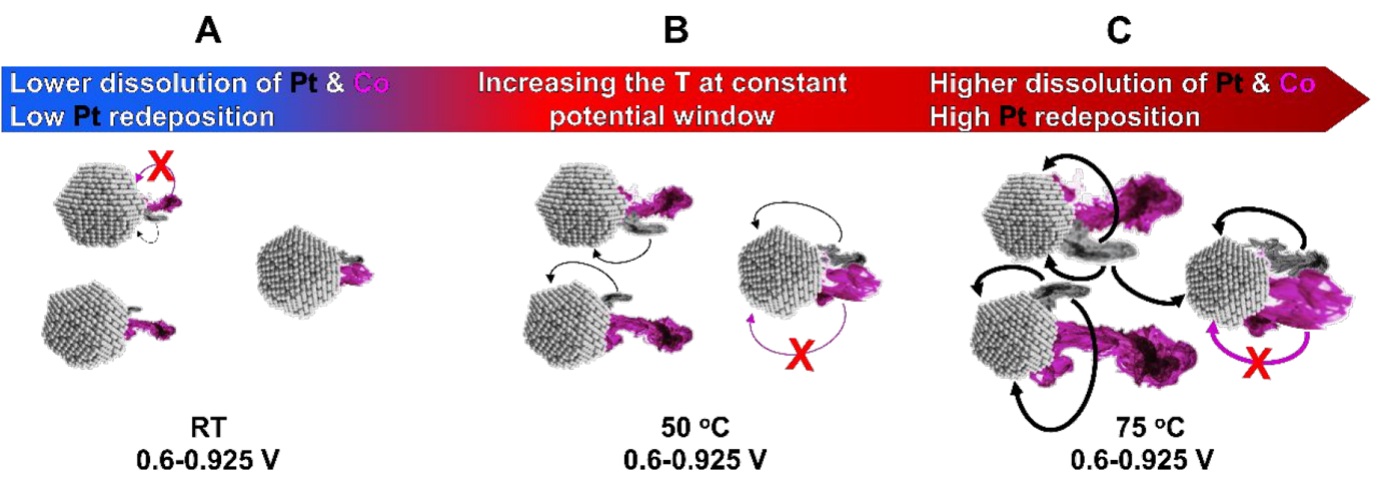
Metal Dissolution Related Degradation Mechanisms Resulting from Increasing the Temperature at a Constant Potential Window Black and magenta mists represent the dissolution of Pt and Co, whereas the arrows indicate on the increasing presence of Pt redeposition.

Figure 4a–c show the LPL effect (3 cycles each LPL, 0.925-*X* V_RHE_; *X* = 0.7, 0.65, 0.6, 5 mV s^–1^, 0.1 M HClO_4_) in relation to the temperature of the electrolyte (RT, 50 and 75 °C). While the details related to the LPL effect will be reported in a separate publication in the near future, briefly at RT (Figure 4a), we notice that at a fixed UPL (in this case 0.925 V_RHE_), upon decreasing the LPL from 0.7 to 0.65 and last to 0.6 V_RHE_, an increase in the dissolution of the less noble metal (in this case Co) is observed. In general, the increase in the observed dissolution of Co in the operational potential window (0.925-*X* V_RHE_; *X* = 0.70, 0.65, 0.60) is predominantly a consequence of the cathodic dissolution of Pt. By decreasing the LPL at a constant UPL, we are reducing increasingly more Pt-oxide during the cathodic scan and, thus, forming more low coordinated Pt atoms that can be dissolved as a result of the oxide-place exchange mechanism.^54^ Once Pt dissolves, it exposes previously protected Co atoms, thus leading to the subsequent dissolution of Co. For now, let us focus on the changes in the trends of signals corresponding to the dissolution of metals (Pt and Co) at various temperatures (Figure 4a–c) and disregard that the observed Pt signal in Figure 4a is in contrast to Co in fact decreasing with each cycle even when we lower the LPL and leave this discussion for the following section. Upon increasing the temperature of the electrolyte to 50 °C (Figure 4b), we notice that in comparison to the RT measurement (Figure 4a), the dissolution of Pt remains very similar or even slightly decreased. Moreover, we notice that the dissolution of Co in fact almost doubled at the LPLs of 0.65 and 0.6 V_RHE_. Before further explanation, let us look at what happens if the temperature is increased to 75 °C (Figure 4c). We notice that the signal corresponding to the dissolution of Pt has nearly dropped into the background (see also SI, Figures S8a–c and S9a–c to observe similar temperature-dependent metal dissolution trends with d-int–Pt-Cu/C and d-int–Pt-Ni/C electrocatalysts). However, rather surprisingly, in contrast to the measurements performed at RT (Figure 4a) and 50 °C (Figure 4b), the dissolution of Co is already significant at the highest LPL of 0.7 V_RHE_. To explain this, we need to consider two things: (i) in accordance to the prior work by Cherevko and co-workers,^42^ increasing temperature shifts the onset of Pt-oxide formation toward lower potentials and the on-set of Pt-oxide reduction toward higher potentials. In other words, this means that at the same potential window (e.g., 0.6–0.925 V) but with increasing electrolyte temperature, one not only forms more Pt-oxide anodically but also reduces more Pt-oxide cathodically, leading to a higher degree of predominantely oxide-place exchange induced dissolution of Pt. Since Pt “protects” the less noble metal, the dissolution of Pt in the case of Pt-alloys is always followed by the dissolution of the less noble metal,^25,26^ a higher degree of Pt dissolution also means more dissolved Co, which is exactly the trend we observe in Figure 4a–c (see also SI, Figures S8a–c and S9a–c to observe similar temperature-dependent metal dissolution trends with d-int–Pt-Cu/C and d-int–Pt-Ni/C electrocatalysts); (ii) since we instead observe a decreasing trend (signal) for Pt dissolution with increasing temperature with the MS detector (Figure 4a–c), this in some way contradicts our previous statement (“a higher degree of Pt dissolution also means more dissolved Co”). This could lead one toward an incorrect conclusion that perhaps Pt is not less stable but might be more stable when increasing temperature. However, as already predicted as a possibility by Cherevko and co-workers,^42^ the observed decrease in the dissolution of Pt with increasing temperature (Figure 4a–c) is in fact a consequence of much more efficient redeposition of Pt. Thus, in reality, under a constant potential window (e.g., 0.6–0.925 V), Pt dissolution and consequently also Co dissolution indeed both increase with increasing operating temperatures (Scheme 2A–C). At the same time, however, an even higher amount of dissolved Pt will redeposit back in the relatively thick (several μm) catalyst layer, most likely for the major part via the Ostwald ripening mechanism and thus, unlike Co, does not reach the ICP-MS detector.^52^ This leads to an impression that we have actually dissolved less Pt rather than more.

At this point, let’s further consider the observed dissolution trends at RT in Figure 4a, namely the observed decreasing trend in Pt dissolution but an increasing trend in Co dissolution when lowering of the LPL at a constant UPL from 0.7 to 0.65 to 0.6 V_RHE_. To further understand this result, a supplementary experiment was performed (see SI, Figure S11) at also RT by fixing the LPL to 0.6 V_RHE_ and increasing the UPL from 0.925 to 1.0 V_RHE_ every three cycles instead. While the amount of information in Figure S11 is astounding by itself, what is simple to observe is that in accordance with expectations, Pt dissolution increases with increasing the UPL.^46^ This can be easily explained by higher UPL correlating with a higher amount of anodically formed Pt-oxide. More Pt-oxide also means more oxide-place exchange and, thus, more dissolved Pt when part of the Pt-oxide gets cathodically reduced.^54^ Furthermore, regardless of the UPL, the Pt surface even at the LPL of 0.6 V_RHE_ is only partly reduced. This all suggests that different Pt-oxides form at various UPLs and that these oxides do not all have the same “stability” and require different LPLs to reduce them. In other words, some Pt-oxides are reduced already at the LPL of 0.7 V_RHE_, some only at 0.6 V_RHE_, and some require an even lower LPL. However, acid washing (dealloying) is already used to prepare the investigated d-int–Pt-Co/C electrocatalyst induces formation of certain native Pt-oxides. When such an electrocatalyst is then exposed again to the acidic environment (0.1 M HClO_4_ electrolyte) in the EFC, we in-fact in these first electrochemical cycles observe the oxide-place exchange induced Pt dissolution resulting from the reduction of these native Pt-oxides. However, because of a rather low UPL of 0.925 V_RHE_, we assume that by going until 0.7/0.65/0.6 V_RHE_, more native Pt-oxide is reduced with each cycle than newly formed electrochemically when going back to 0.925 V_RHE_. Since Pt is also always redepositing (even at RT – Scheme 2A) but Co does not, there is always some Pt that does not reach the MS detector, and correspondingly, the observed Pt signal in Figure 4a decreases with each cycle. Because the observed Pt signals are already in the range of 1 ppb even when enough native Pt-oxide is formed via acid washing, it becomes now clear that the future flow cell experiments could benefit from using UPLs between 0.95 and 1.0 V_RHE_ instead of 0.925 V_RHE_. As seen in the supplementary experiment in Figure S11, this would increase the formation of electrochemically formed Pt-oxide with each anodic scan and bring Pt dissolution signals above the limits of detection despite redeposition of Pt. Nevertheless, since the present study focuses on Pt-alloys, by using the less noble metal as a probe still provides for a rare opportunity to look “beyond the limits of detection” and connect the “invisible” Pt signal with the visible Co signal.

With respect to temperature dependence, Figure 4d–f show the wide potential window (WPW) cycles (III.; two WPW cycles, 0.05–1.4 V_RHE_, 10 mV s^–1^, 0.1 M HClO_4_). Here we wish to remind the reader that each of the six measurements in Figure 4 was performed on a fresh catalyst film, thus avoiding any memory effects. In contrast to the LPL experiments (Figure 4a–c), the WPW experiments (Figure 4d–f) are more easily relatable to the prior work by Cherevko and co-workers.^42^ Similarly to their work, with increasing temperature, we observe a decrease in the signal related to the cathodic dissolution of Pt as well as a decrease in the shift of the peak maximum toward higher potentials. This is in accordance with two already explained phenomena – (i) shift in the cathodic peak maximum of Pt due to the shift in the on-set of Pt-oxide reduction toward higher potentials and (ii) a decrease in the signal corresponding to the cathodic dissolution of Pt due to a higher degree of Pt redeposition with increasing temperature. Furthermore, in contrast to the LPL experiments (Figure 4a–c), in the case of the WPW experiments (Figure 4d–f), we observe only minor differences in the dissolution of Co with increasing temperature. This is because unlike in the case of LPL experiments (Figure 4a–c), where only a part of the Pt-oxide gets reduced upon the cathodic scan until 0.7, 0.65, or 0.6 V_RHE_, by going as low as 0.05 V_RHE_ as in the case of WPW experiments (Figure 4a-c) Pt-oxide gets reduced entirely regardless of the temperature. Consequently, any differences related to Co dissolution at wide potential windows become less significant, or in other words, the effect of temperature on the stability of Pt-alloys is perhaps much more important in the case of PEMFC operational voltage window (i.e., 0.6–0.95 V).

**Figure 5:**
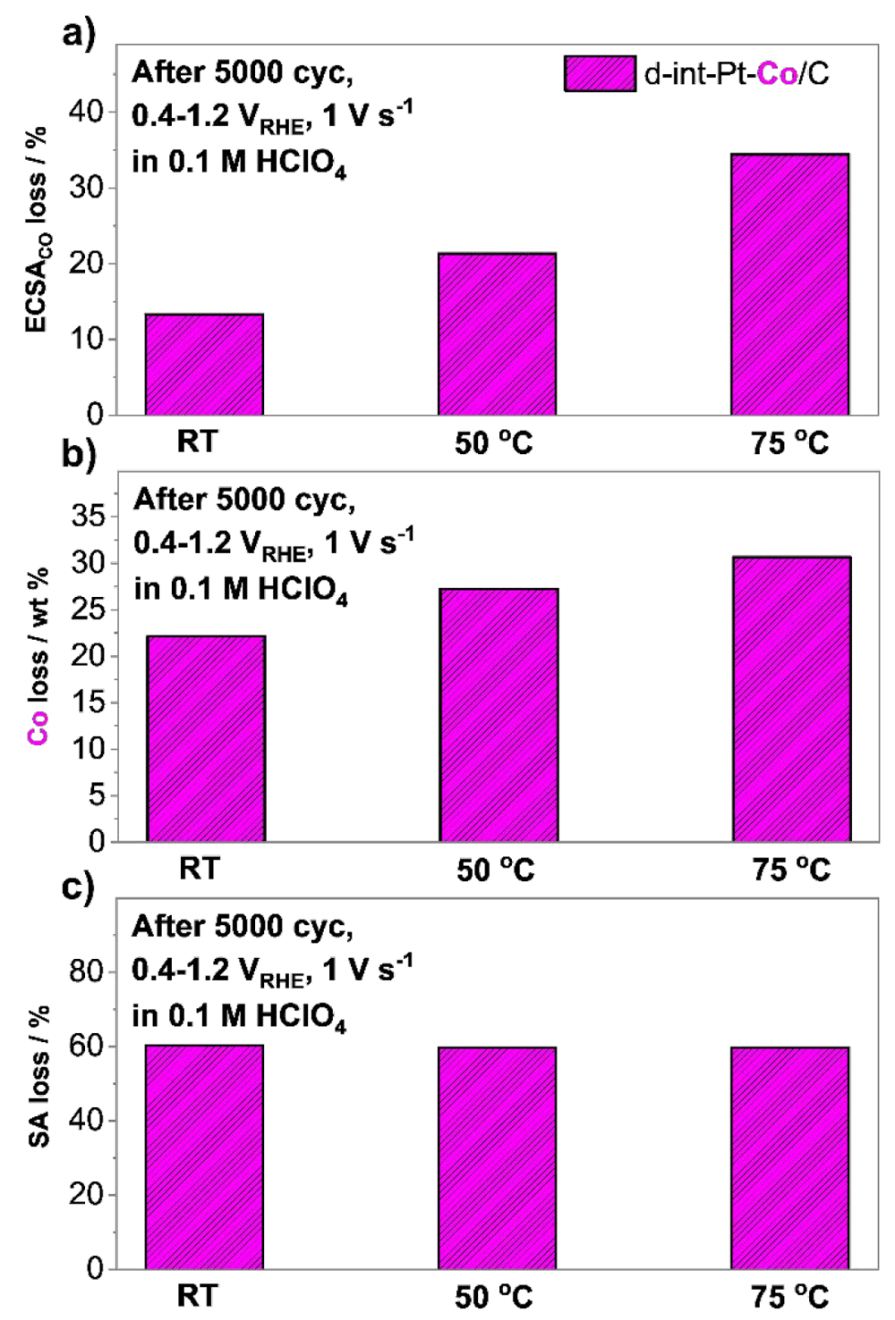
Effect of the temperature on the accelerated degradation tests (ADTs) performed at a constant potential window (0.4–1.2 V_RHE_, 5000 cycles, 1 V s^–1^, 0.1 M HClO_4_) comparing (a) ECSA_CO_ loss, (b) SA loss, and (c) Co loss of the ReCatalyst intermetallic dealloyed Pt–Co/C electrocatalyst. Electrochemical data along with a comparison with Elyst Pt50 0690 benchmark from Umicore can be found in SI, Figures S12–S13.

Nevertheless, let us also look at the effect of temperature when using harsher ADT conditions, Thus, for the final assessment (Figure 5), we compare the ReCatalyst electrocatalyst at various temperatures (RT, 50 and 75 °C) at a constant but wider potential window (0.4–1.2 V_RHE_, 5000 cycles, 1 V s^–1^, 0.1 M HClO_4_). While these conditions are unrealistic in terms of the expected operational conditions of a PEMFC (i.e., 0.6–0.95 V), they resemble the harsher conditions used during our previous assessment of Pt/C electrocatalysts from Tanaka Kikinzoku Kogyo used for the validation of the in-house designed HT-DE methodology (UPL of 1.2 V at 75 °C) in our previous work.^31^ However, the main focus of this set of experiments is to provide evidence that in addition to the expected increased contribution of corrosion of the carbon support with increasing temperature, also under harsher ADT conditions, the contribution of Pt dissolution still holds a very relevant significance to the overall degradation of carbon supported Pt-alloy electrocatalysts investigated in the present study. Figure 5a presents the expected ECSA_CO_ loss trend with increasing temperature that follows RT < 50 °C < 75 °C. By looking at both the past literature and our prior work that assessed only pure-Pt systems, such a trend could lead to the incomplete assumption that an increase in ECSA_CO_ loss with increasing temperature is dominated by the increased corrosion of the carbon support, followed by coalescence and agglomeration of Pt-based NPs and/or their detachment.^31−33^ However, evidence in the present work (Figures 2–4) suggests that increasing the temperature under a constant potential window results in a combination of both an increased dissolution of Pt as well as an increased rate of Pt redeposition (Scheme 2A–C) predominantly via the Ostwald ripening mechanism. This is in line also with the observations from our recent temperature-dependent electrochemical modeling study where an increase in Ostwald ripening was shown as the dominant mechanism to significantly increase with increasing temperature.^52^ Similarly to all other experiments, also here the Co loss presented in Figure 5b increases with increasing temperature and thus follows a similar trend as ECSA_CO_ – RT < 50 °C < 75 °C. This is in line with the statement that even under the ADT conditions presented in Figure 5, with increasing temperature, corrosion of the carbon support is responsible only for a part of the observed ECSA_CO_ loss and that the contribution of Pt dissolution is again far from negligible. To further support this, we have performed additional ex situ HAADF and BF STEM imaging (see SI, Figure S14) of ensembles of Pt–Co NPs after the ADT at 75 °C as well as carefully selected representative cases of individual intermetallic Pt–Co NPs to provide a comparison of their structure before and after such an ADT (see SI, Figure S15). The collected data on ensembles of NPs suggests degradation mechanisms resulting from both corrosion of the carbon support (e.g., necking) as well as metal dissolution (e.g., thickening of the Pt-rich shells). To further discuss the present data from Figures S14 and S15, several prior studies on the “disordered” *Fm*3̅*m* Pt–Co NPs presented clear evidence on the formation of “hollow” NPs resulting from presumably the Kirkendall effect—faster transport of Co atoms in contrast to Pt from the core of the NPs toward the surface, followed by its dissolution.^77^ In contrast to these prior studies, the present data (see SI, Figures S14 and S15), despite the rather harsh ADT conditions, provides evidence that the observed “ordered” Pt-alloy NPs retain both the intermetallic crystal lattice in the core as well as the core–shell structure (with a thicker Pt-rich shell). While this goes beyond the scope of the present study and requires a deeper investigation, the implications of these observations are rather significant. Specifically, this suggests that perhaps an “ordered” intermetallic structure holds a significant importance at slowing down the transport of Co from the core of the NPs toward the surface; this limits both the formation of any “hollow” features and consequently significant Co dissolution arising from the Pt-alloy core.

Lastly, in contrast to the temperature comparison in the case of the potential window of 0.6–1.0 V_RHE_ (Figure 3), in the case of the potential window of 0.4–1.2 V_RHE_, the loss in SA for ORR does not follow any specific trends such as ECSA_CO_ and Co loss and seems to be similar regardless of the temperature used in the ADT (Figure 5c). One could explain that regardless of the temperature, when performing as much as 5000 cycles under the given ADT conditions, the Pt–Co intermetallic NPs eventually reach a similar quasi-stable state, resulting in the loss of SA from the decrease of the strain and the ligand effects, resulting from the observed depletion and thickening of the Pt-rich shell (see SI, Figure S15).^79,80^ However, most likely in the case of performing the ADT at 75 °C, one reaches such a state after a significantly lower number of cycles. Thus, if the operation of Pt-alloys is not limited to a narrow voltage window, one can eventually expect significant kinetic performance losses for ORR regardless of the operating temperature.

## Conclusions

While the rate of carbon corrosion follows the Arrhenius law and increases exponentially with temperature, the findings of the present study contradict the generally accepted hypothesis that the kinetics of Pt and subsequently the less noble metal dissolution are supposed to be for the most part unaffected by temperature.^81^ However, clear evidence is presented that in addition to the importance of the voltage/potential window,^31,71^ temperature is in fact the most critical parameter governing the stability of Pt and thus in the case of Pt-alloy electrocatalysts also the ability of the NPs to retain the less noble metal. In addition, based on the mechanistic insights obtained with the HT-EFC-ICP-MS, the findings of this study also provide evidence that the observations by Cherevko and co-workers^42^ on polycrystalline Pt are indeed a consequence of severe Pt redeposition at increased temperature. This also hints that at elevated temperatures, carbon-supported Pt-based electrocatalysts degrade also via the Ostwald ripening mechanism and not just the carbon corrosion-induced Pt agglomeration. The results can be summarized in the following main messages:

The HT-DE methodology revealed that a higher temperature of the electrolyte (0.1 M HClO_4_ in the present study) will result in an increased loss of ECSA_CO_, SA as well as an increase in the loss of Co. At the same temperature (e.g., 75 °C), this is also true when one expands the potential window—both the lower or the upper potential limit. While the relation seems to be rather linear for upper potential limits of 1.0 V or below, it becomes exponential if the potential is to be increased to 1.2 V (or higher).
Evaluating the stability of novel Pt-alloy electrocatalysts at RT and in the potential window of 0.6–1 V is insufficient. This work provides evidence that performing ADTs under such a potential window at 75 °C, we observed not only a noticeably higher loss of ECSA_CO_ and SA but also a higher loss of the less noble metal. While this additional dissolved Co in the present study gets diluted in a large amount of the electrolyte, one can expect a significantly higher impact if such an amount of Co would be dissolved in a fuel cell.^61,82^ In addition, further narrowing of the operational window (e.g., 0.7–0.925 V) results in a significant decrease in the detected amounts of dissolved Co.
The HT-EFC-ICP-MS methodology, however, revealed that most likely, different dynamics of Pt-oxide formation and reduction at elevated temperature conditions^42^ are responsible for the increased dissolution of Pt with increasing temperature. At the same potential window, both more Pt-oxide is formed during the anodic part of the scan as well as reduced during the cathodic part, resulting in more transiently dissolved Pt predominantly as a result of the oxide-place exchange mechanism. Nevertheless, this increase in Pt dissolution is masked by also a significant increase in the Pt redeposition in the catalyst layer. However, because less noble metal dissolution (dealloying) is a consequence of Pt dissolution,^26^ the observed increase in the less noble metal dissolution with increasing temperature provides evidence of what is going on with Pt. Thus, a significant increase in the cathodic less noble metal dissolution already in the potential window of 0.7–0.925 V is observed with increasing temperature.

This work provides a significant contribution toward lowering of the so-far highly speculative mechanistic interpretation of the temperature and potential window-dependent kinetics of metal dissolution and the likely mechanisms behind them. In addition, the work shows significant evidence that while intermetallic alloys of Pt are quite stable when used under rather narrow operational windows/voltages, they do not stop the leaching of the less noble metal under none of the used ADT conditions. Nevertheless, this work not only provides guidance to the PEMFC community on the investigation of the stability of novel Pt-alloy electrocatalysts but also adds value to the importance of designing a more stable catalyst layer that accounts for redeposition and operating voltage window. For widespread deployment of Pt-alloy electrocatalysts in the industry, it also holds valuable information to the system-level producers who need to provide for crucial hardware and/or software solutions to limit the operating voltage as well.

## Supporting Information Available

HT-DE scheme, HT-EFC-ICP-MS setup image, TEM images of catalysts, electrochemical comparison of catalysts (ORR and CO-electrooxidation), electrochemical comparison before and after various ADTs (ORR and CO-electrooxidation), HT-EFC-ICP-MS measurements of d–Pt-Ni/C and d–Pt-Cu/C catalysts, HT-EFC-ICP-MS reproducibility (PDF)
