# Supplementary material for: Understanding the Crucial Significance of the Temperature and Potential Window on the Stability of Carbon Supported Pt-Alloy Nanoparticles as Oxygen Reduction Reaction Electrocatalysts
Source: ACS Catal. 2021 Dec 13;12(1):101–15. doi: 10.1021/acscatal.1c04205 (PMC8749953; doi:10.1021/acscatal.1c04205)
Supplement: Supplementary file 1 — cs1c04205_si_001.pdf [file cs1c04205_si_001.pdf]

Supporting information: Understanding the Crucial Significance of the Temperature and Potential Window on the Stability of Carbon Supported Pt-alloy Nanoparticles as Oxygen Reduction Reaction Electrocatalysts

Tina Đukić[a,b‡], Leonard Jean Moriau[a‡], Luka Pavko[a,b], Mitja Kostelec[a,b], Martin Prokop[c], Francisco Ruiz-Zepeda[a], Martin Šala[d], Goran Dražić[a], Matija Gatalo[a,e*], Nejc Hodnik[a,f]

[a] Department of Materials Chemistry, National Institute of Chemistry, Hajdrihova 19, 1001 Ljubljana, Slovenia

[b] Faculty of Chemistry and Chemical Technology, University of Ljubljana, Večna pot 113, 1000 Ljubljana, Slovenia

[c] University of Chemistry and Technology Prague, Technická 5, 166 28 Prague 6 – Dejvice, Czech Republic

[d] Department of Analytical Chemistry, National Institute of Chemistry, Hajdrihova 19, 1001 Ljubljana, Slovenia

[e] ReCatalyst d.o.o., Hajdrihova 19, 1001 Ljubljana, Slovenia

[f] University of Nova Gorica, Vipavska 13, 5000 Nova Gorica, Slovenia

‡ these authors contributed equally to this work

* to whom correspondence should be addressed: matija.gatalo@ki.si, nejc.hodnik@ki.si

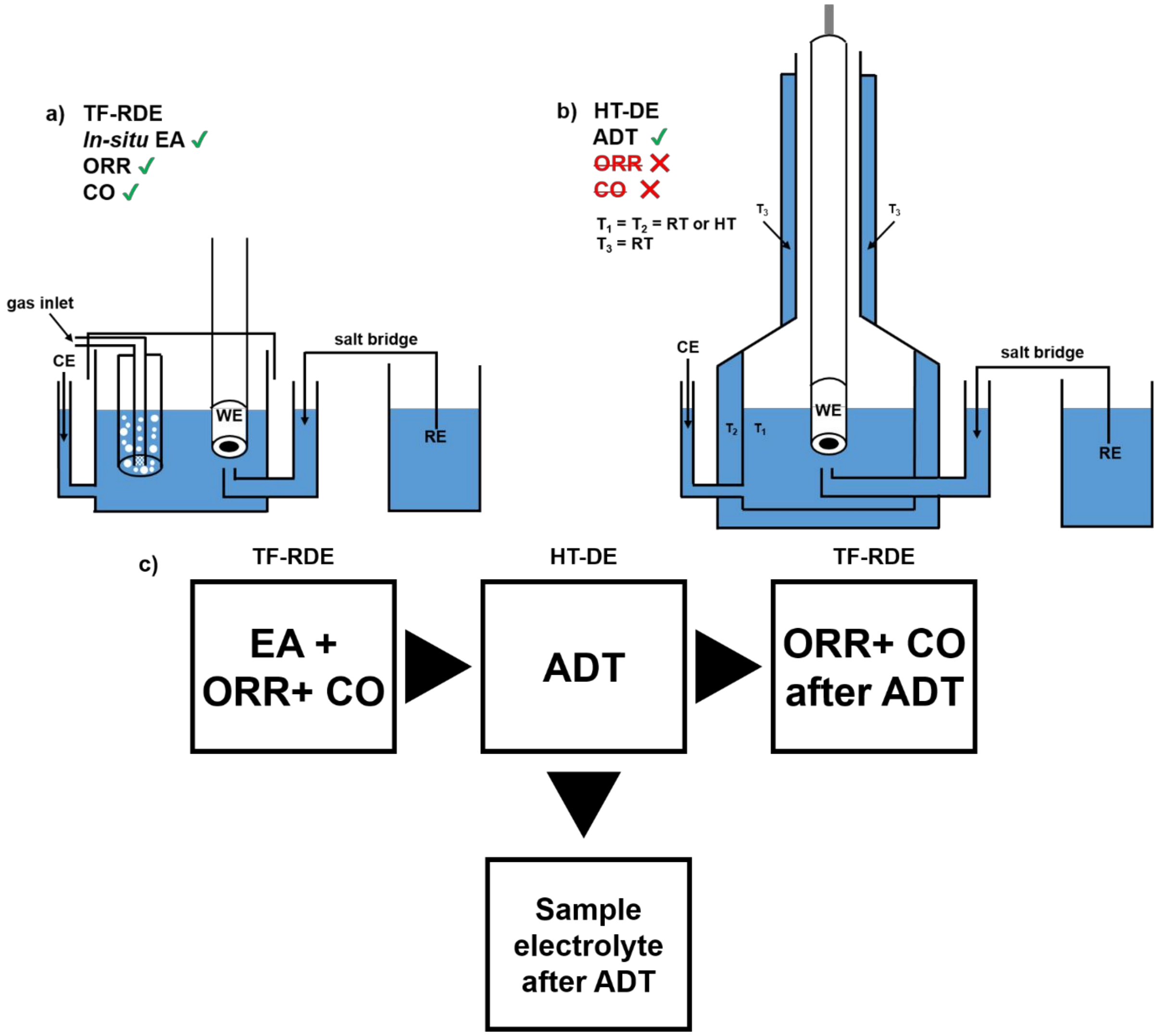

Figure S1: (a-b) Schemes of both the TF-RDE and HT-DE [1] setups used to evaluate the properties of electrocatalysts within this study as well as perform the ADTs at various temperatures and potential windows. **(c)** Box-chart of the experimental flow used to evaluate the electrocatalysts at various potential windows and temperatures.

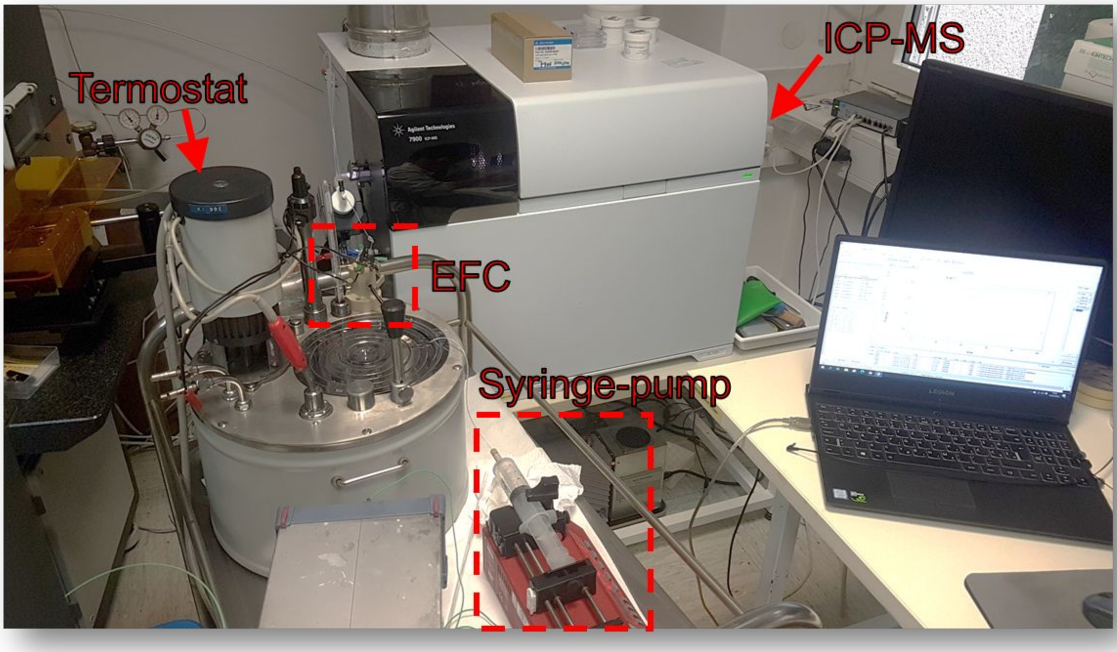

Figure S2: Image of the actual HT-EFC-ICP-MS setup as shown in the **Scheme 1** of the main manuscript.

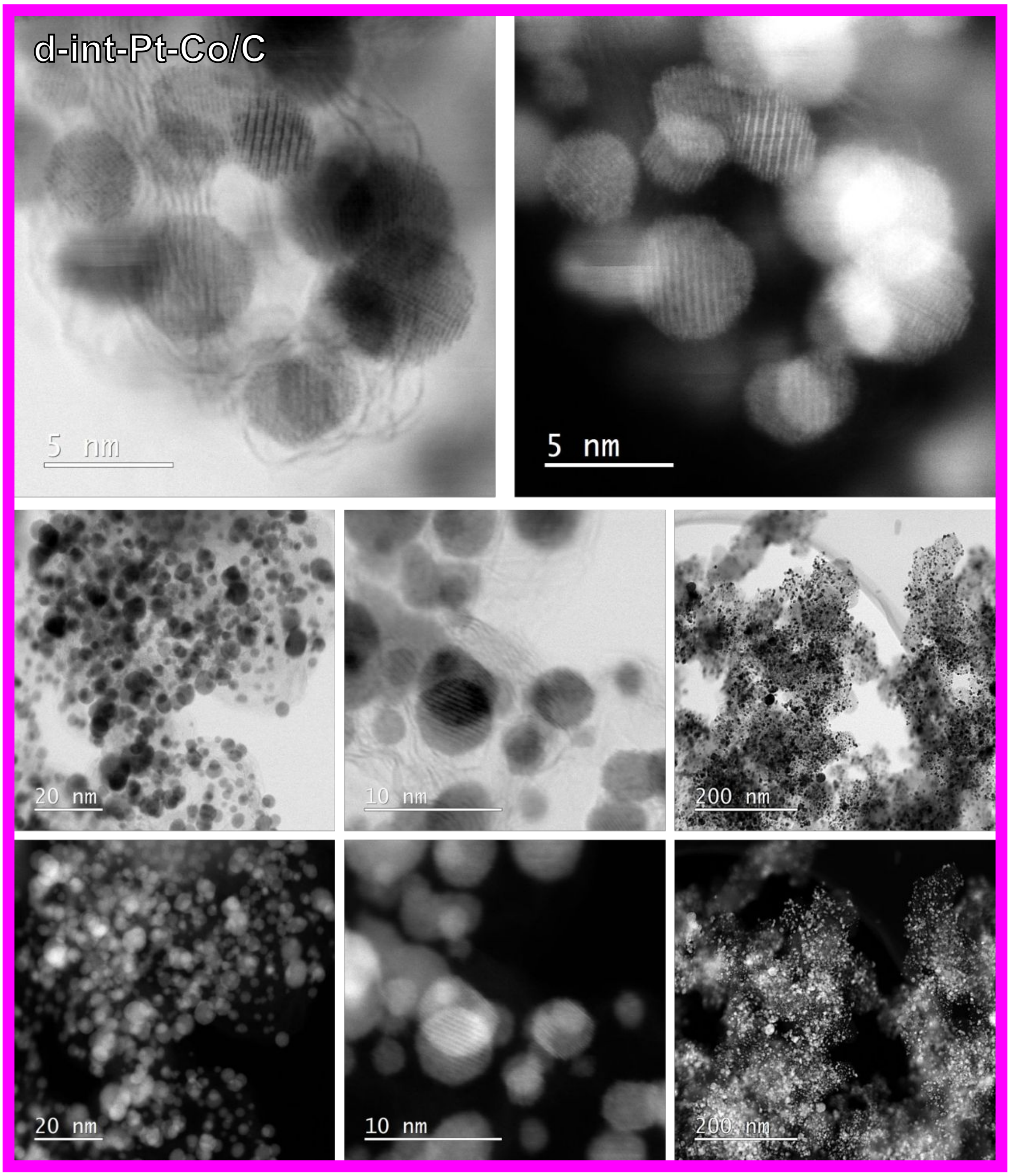

Figure S3: STEM and BF images of the experimental d-int-Pt-Co/C ReCatalyst electrocatalyst at various magnifications. In all Figures, magenta is used for the data corresponding to the experimental ReCatalyst electrocatalyst.

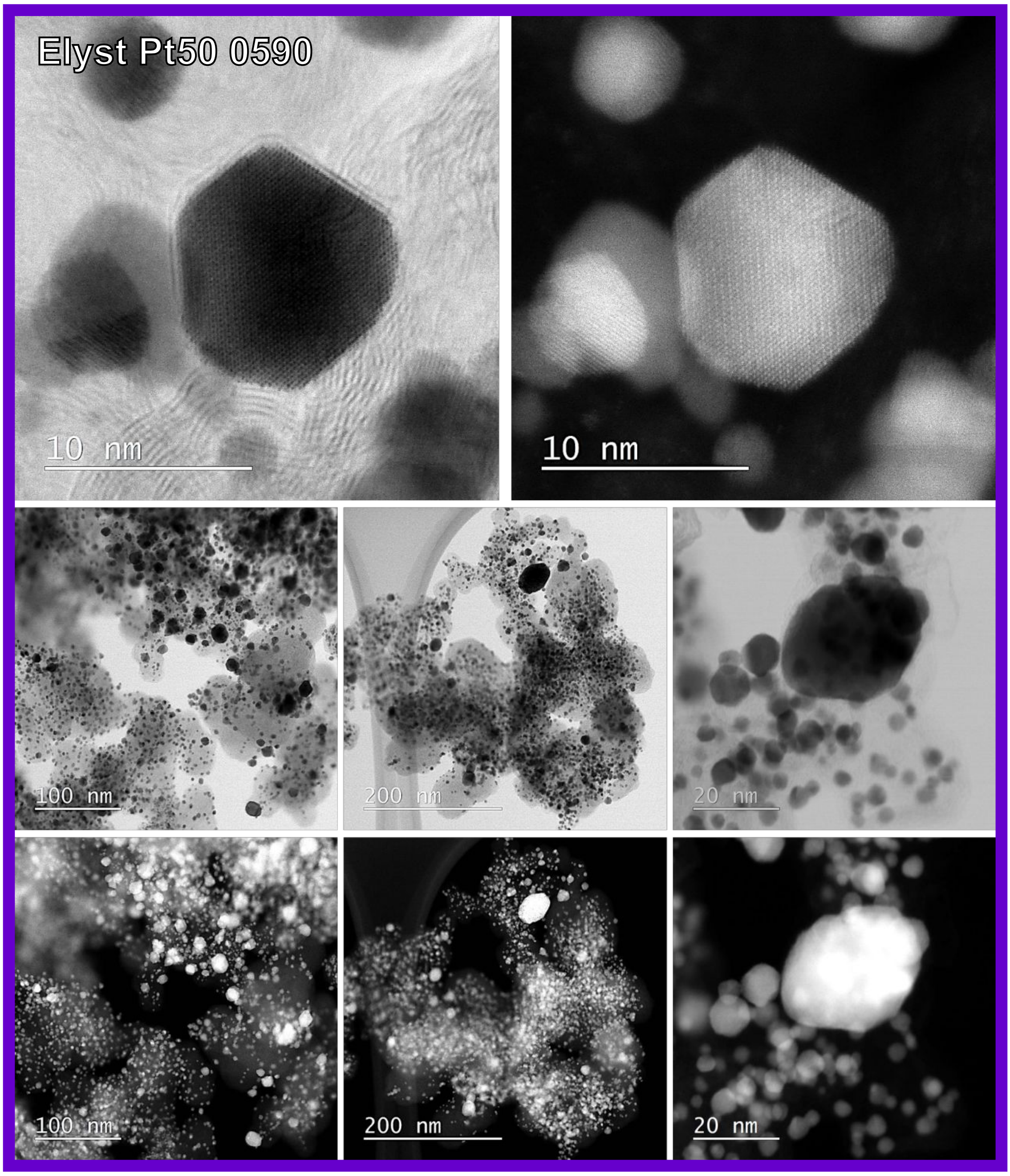

Figure S4: STEM and BF images of the commercially available Pt-Co/C benchamrk electrocatalyst from Umicore (Elyst Pt50 0690) at various magnifications. In all Figures, purple is used for the data corresponding to the Umicore Pt-Co benchmark.

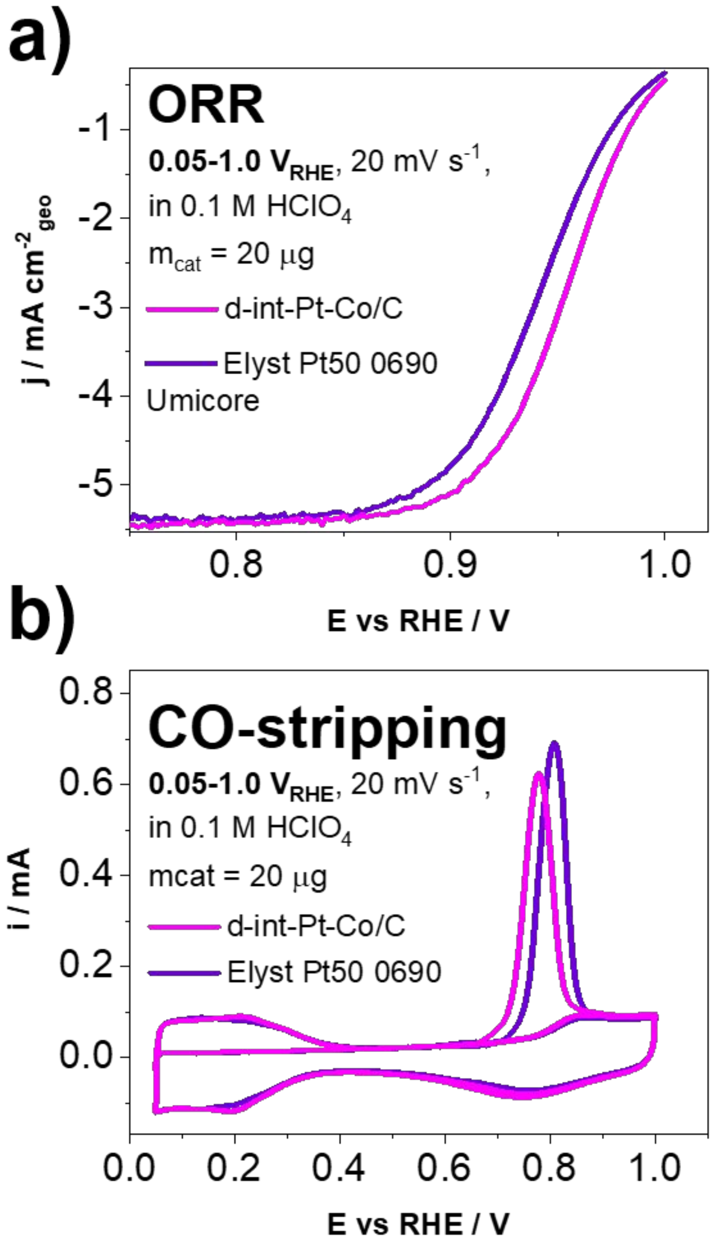

Figure S5: Comparison of **(a)** ORR polarisation curves as well as **(b)** CO-electrooxidation ('stripping') CVs of both the experimental d-int-Pt-Co/C as well as Elyst Pt50 0690 electrocatalysts. In all Figures, magenta is used for the data corresponding to the experimental ReCatalyst electrocatalyst, whereas the data corresponding to the Umicore Pt-Co benchmark is in purple.

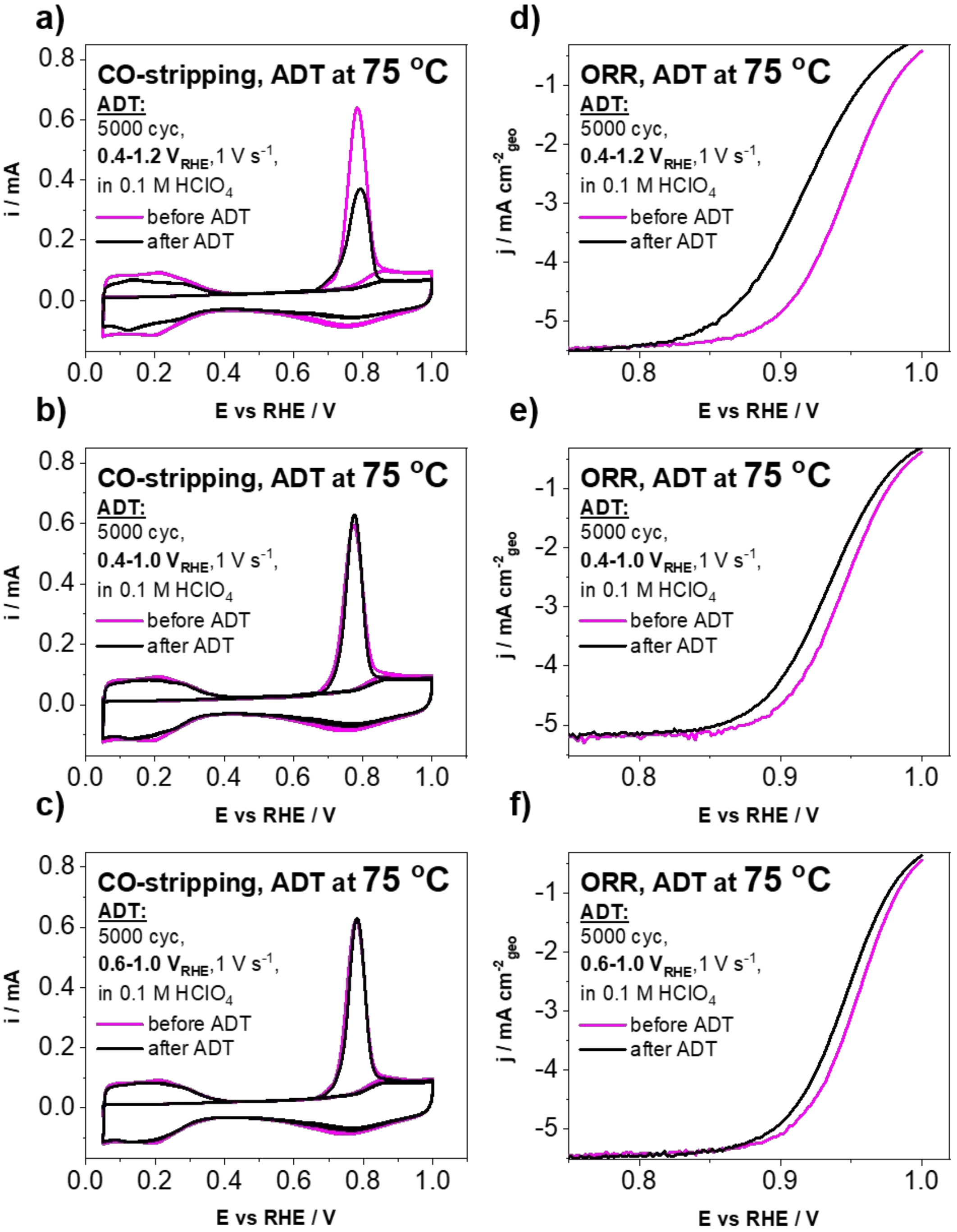

Figure S6: Comparison of **(a-c)** the CO-electrooxidation CVs as well as the follow-up cycles and **(d-f)** ORR polarization curves before and after the ADT performed at 75 °C and various potential windows (5000 cycles, 1 V s^{-1}, 0.1 M $HClO_4$) for the experimental Pt-Co ReCatalyst electrocatalyst.

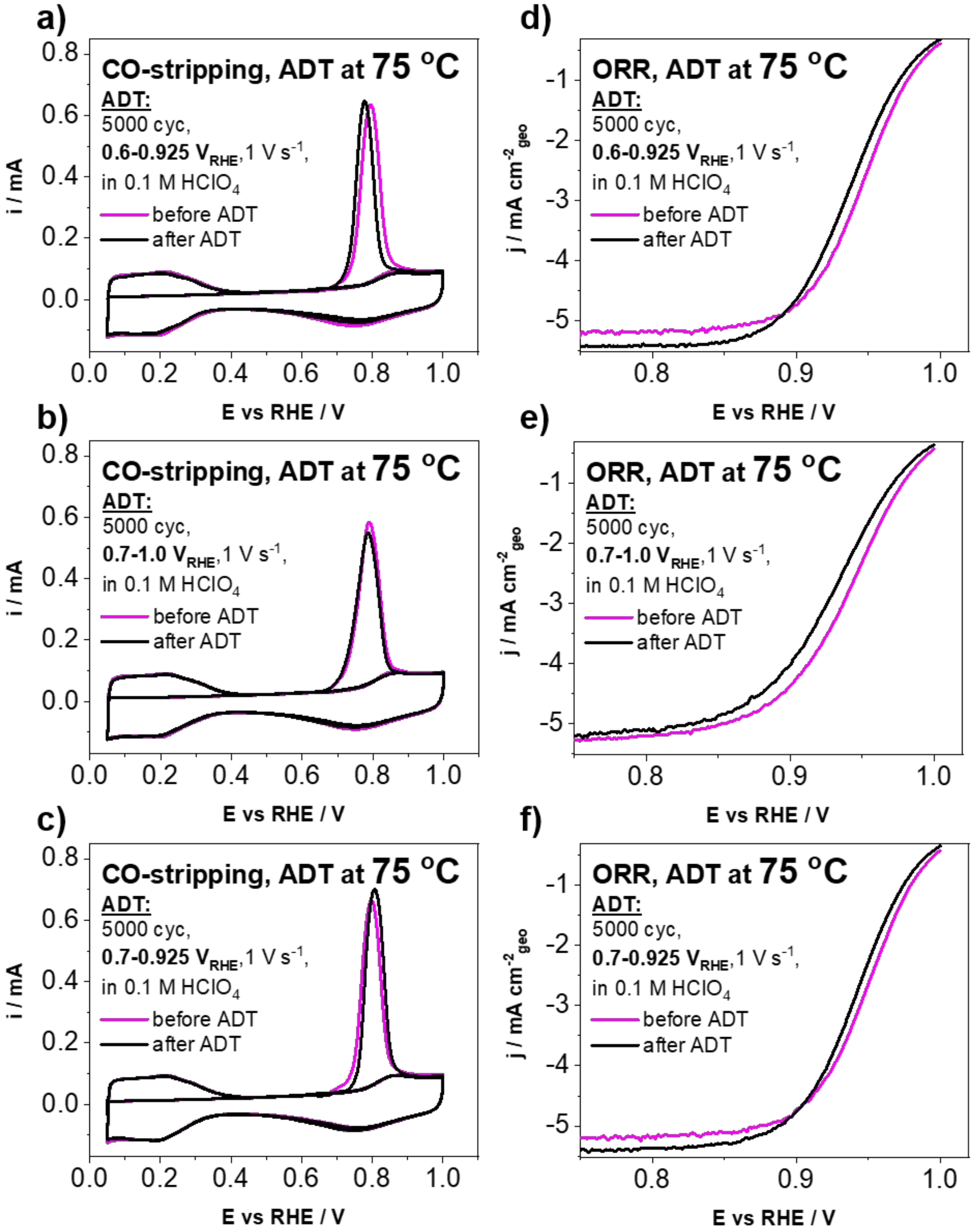

Figure S7: Comparison of **(a-c)** the CO-electrooxidation CVs as well as the follow-up cycles and **(d-f)** ORR polarization curves before and after the ADT performed at 75 °C and various potential windows (5000 cycles, 1 V s^{-1}, 0.1 M $HClO_4$) for the experimental Pt-Co ReCatalyst electrocatalyst.

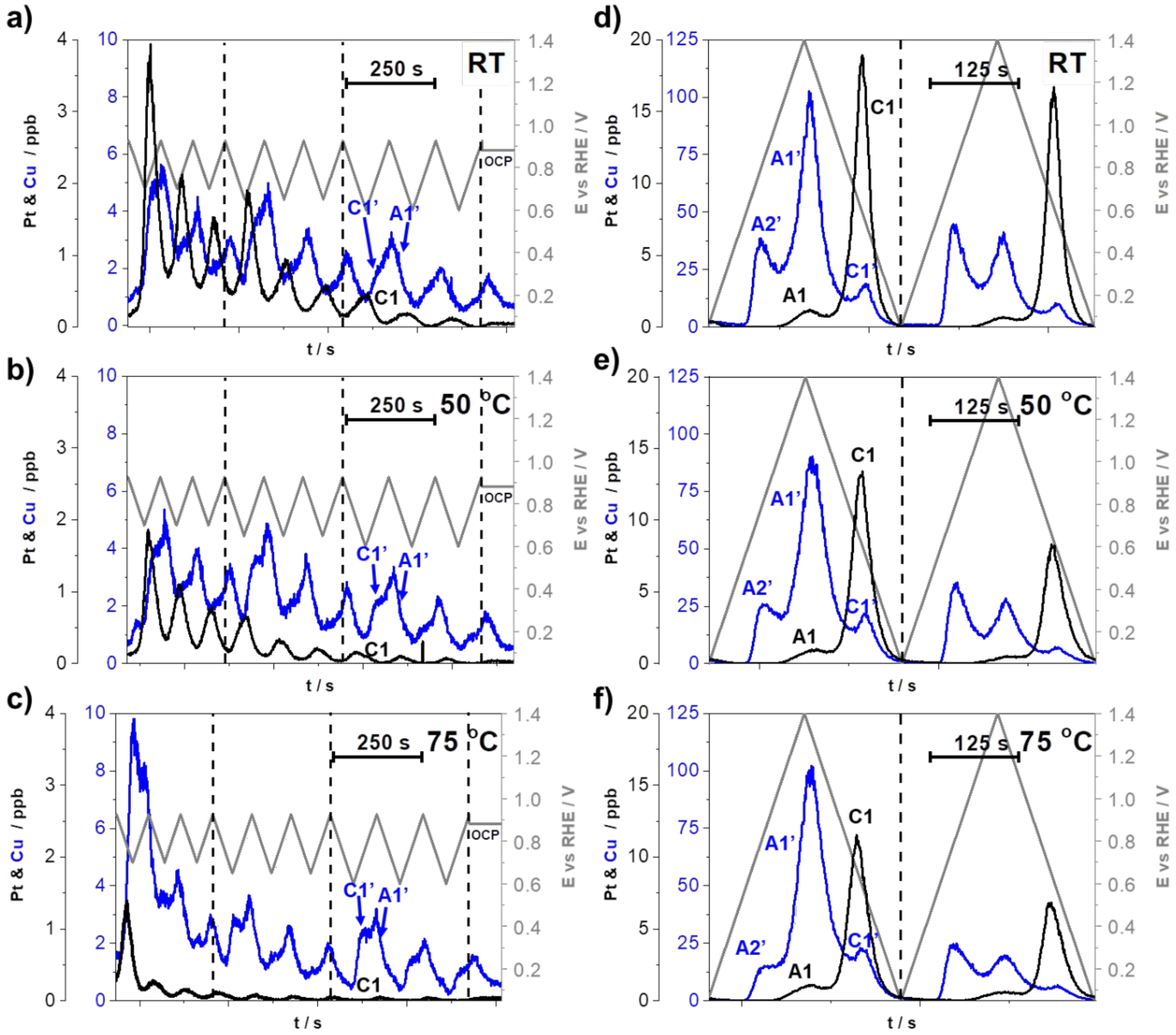

Figure S8: Demonstration of the effect of temperature (RT, 50 ^{o}C, 75 ^{o}C) **(a-c)** on the metal dissolution (Pt & Cu) during the LPL cycles (0.925-X V_{RHE}; X = 0.7, 0.65 and 0.6; 5 mV s^{-1}) and **(d-f)** WPW cycles (two cycles between 0.05-1.4 V_{RHE}, 10 mV s^{-1}) using the HT-EFC-ICP-MS setup in the flow of 0.1 M $HClO_4$. Each metal has its own Y axis to better compare the profiles despite the detected concentration differences. A1, A1', A2 and A2' represent peaks corresponding to anodic dissolution, whereas C1, C1', C2 and C2' represent peaks corresponding to the cathodic dissolution of Pt and Cu respectively. The gray zig zag line represents the cycles from LPL to UPL. Transition between different series of cycles is denoted by the dashed lines.

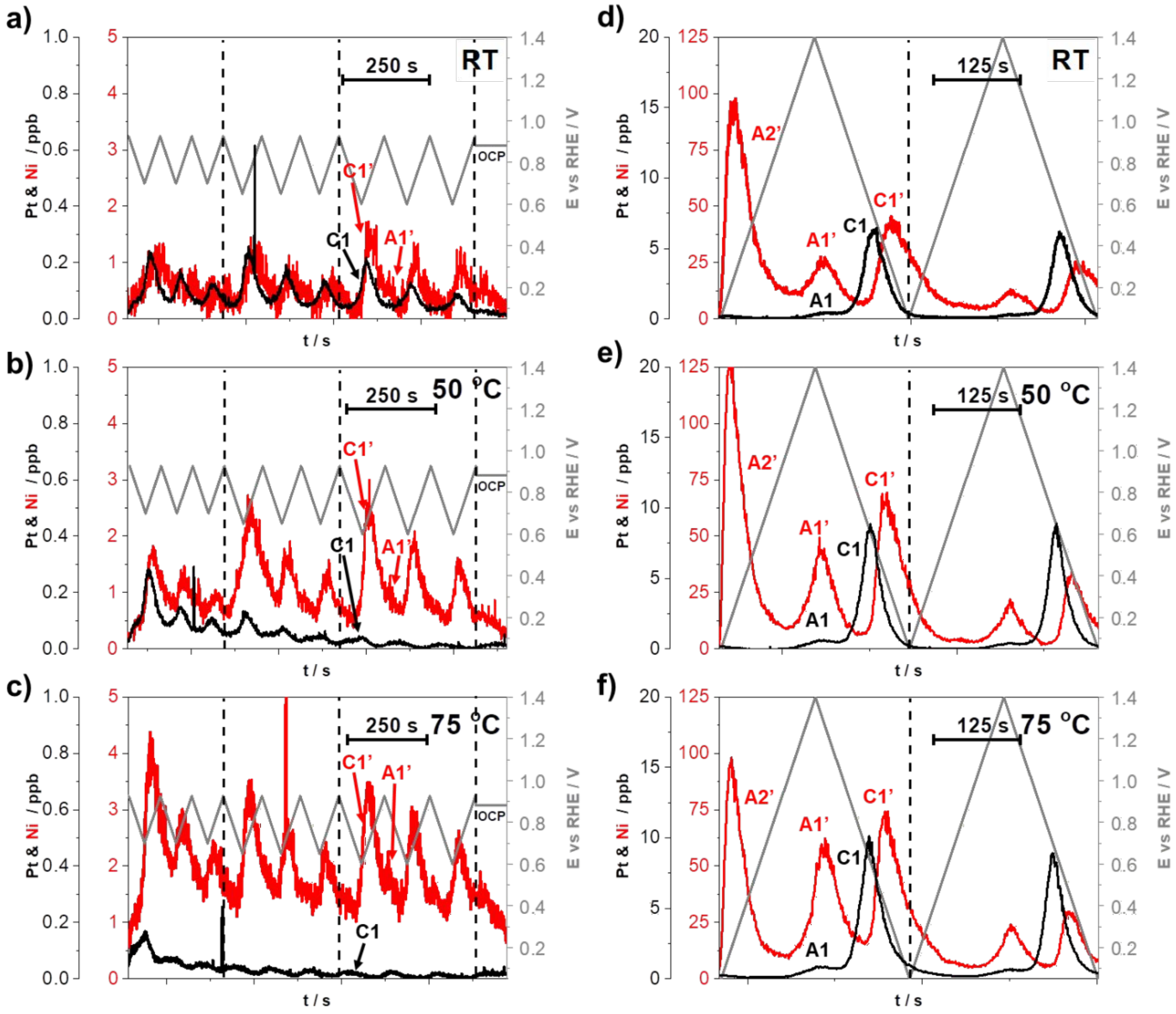

Figure S9: Demonstration of the effect of temperature (RT, 50 ^{o}C, 75 ^{o}C) **(a-c)** on the metal dissolution (Pt & Ni) during the LPL cycles (0.925-X V_{RHE}; X = 0.7, 0.65 and 0.6; 5 mV s^{-1}) and **(d-f)** WPW cycles (two cycles between 0.05-1.4 V_{RHE}, 10 mV s^{-1}) using the HT-EFC-ICP-MS setup in the flow of 0.1 M $HClO_4$. Each metal has its own Y axis to better compare the profiles despite the detected concentration differences. A1, A1', A2 and A2' represent peaks corresponding to anodic dissolution, whereas C1, C1', C2 and C2' represent peaks corresponding to the cathodic dissolution of Pt and Ni respectively. The gray zig zag line represents the cycles from LPL to UPL. Transition between different series of cycles is denoted by the dashed lines.

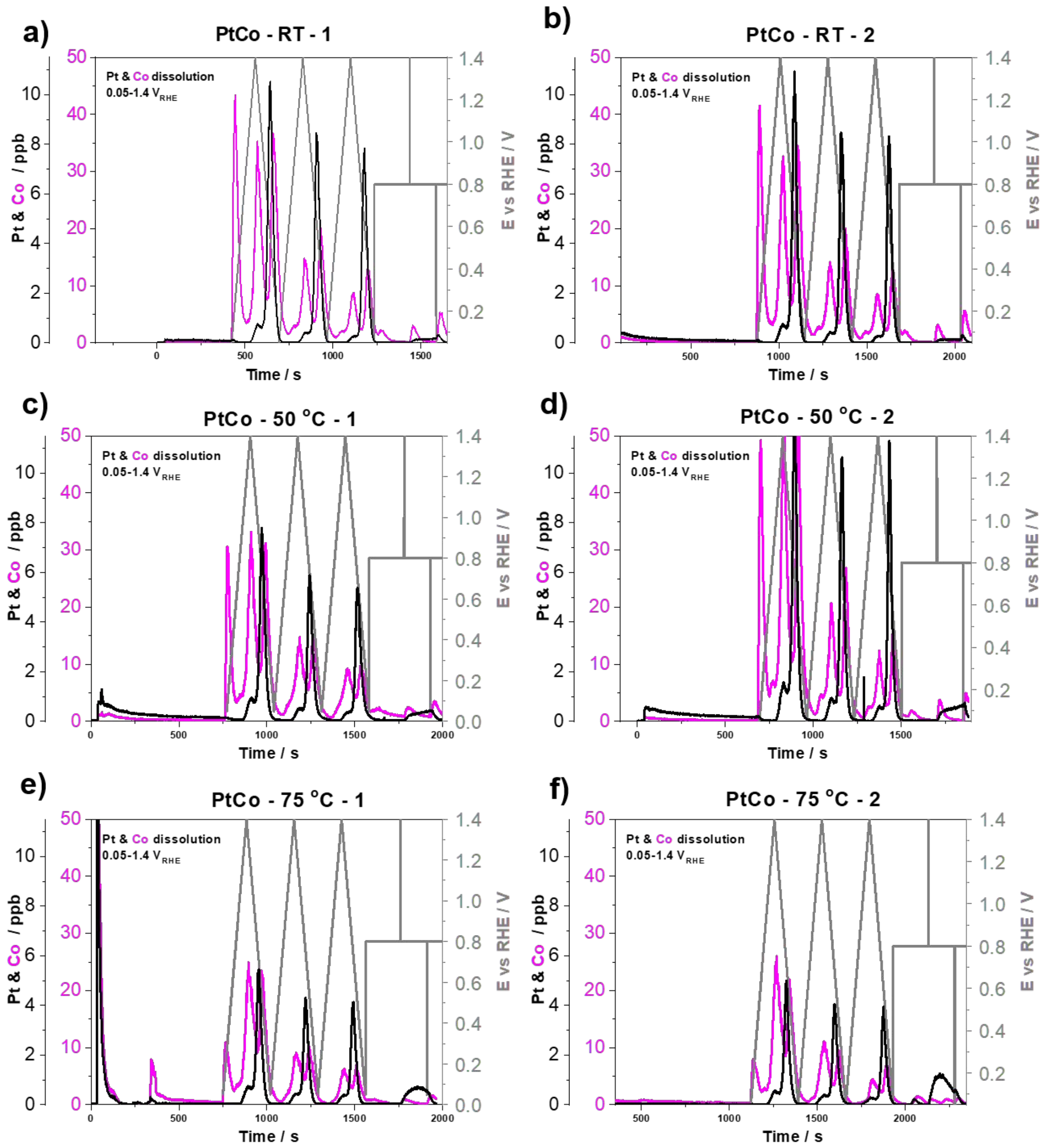

Figure S10: Comparison of two measurements at each temperature (RT – **a,b**; 50 °C – **c,d**; 75 °C – **e,f**) for the WPW cycles (0.05-1.4 V_{RHE}, 10 mV s^{-1}) showing the reproducibility of the results when using the HT-EFC-ICP-MS setup in the flow of 0.1 M $HClO_4$.

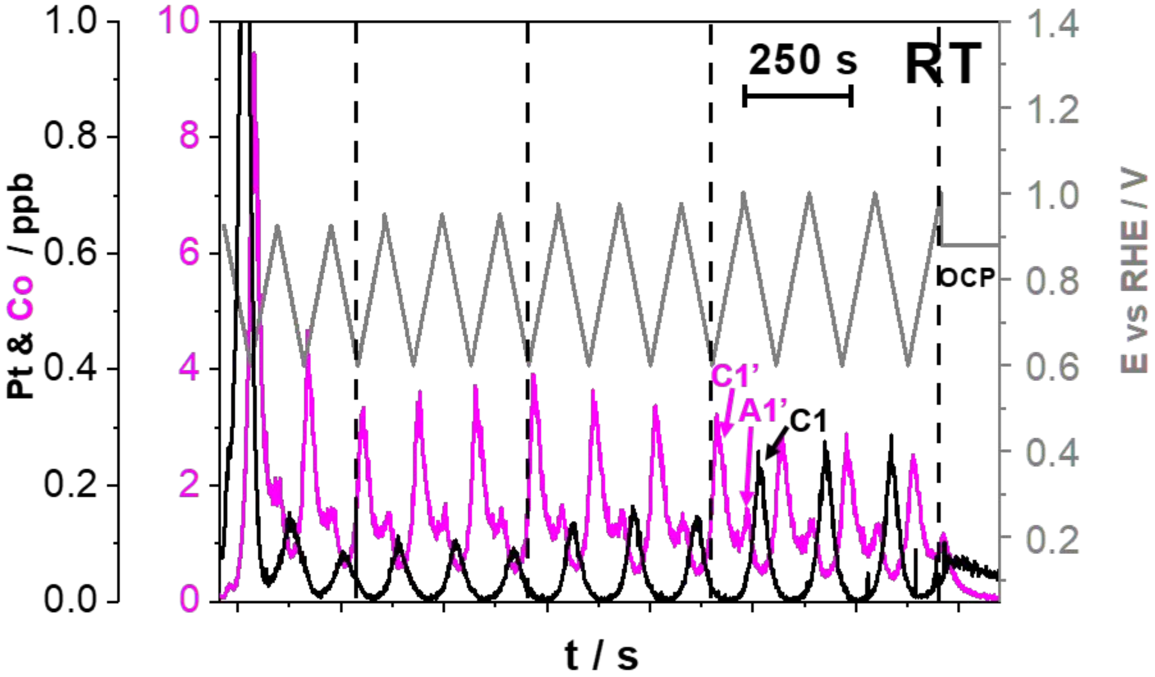

Figure S11: Effect of increasing the UPL at constant LPL on the metal dissolution (Pt & Co) (X-0.6 V_{RHE}; X = 0.925, 0.95, 0.975 and 1.0; 5 mV s^{-1}) at RT demonstrated using the HT-EFC-ICP-MS setup in the flow of 0.1 M $HClO_4$. Each metal has its own Y axis to better compare the profiles despite the detected concentration differences. A1, A1', A2 and A2' represent peaks corresponding to anodic dissolution, whereas C1, C1', C2 and C2' represent peaks corresponding to the cathodic dissolution of Pt and Co respectively. The gray zig zag line represents the cycles from LPL to UPL. Transition between different series of cycles is denoted by the dashed lines.

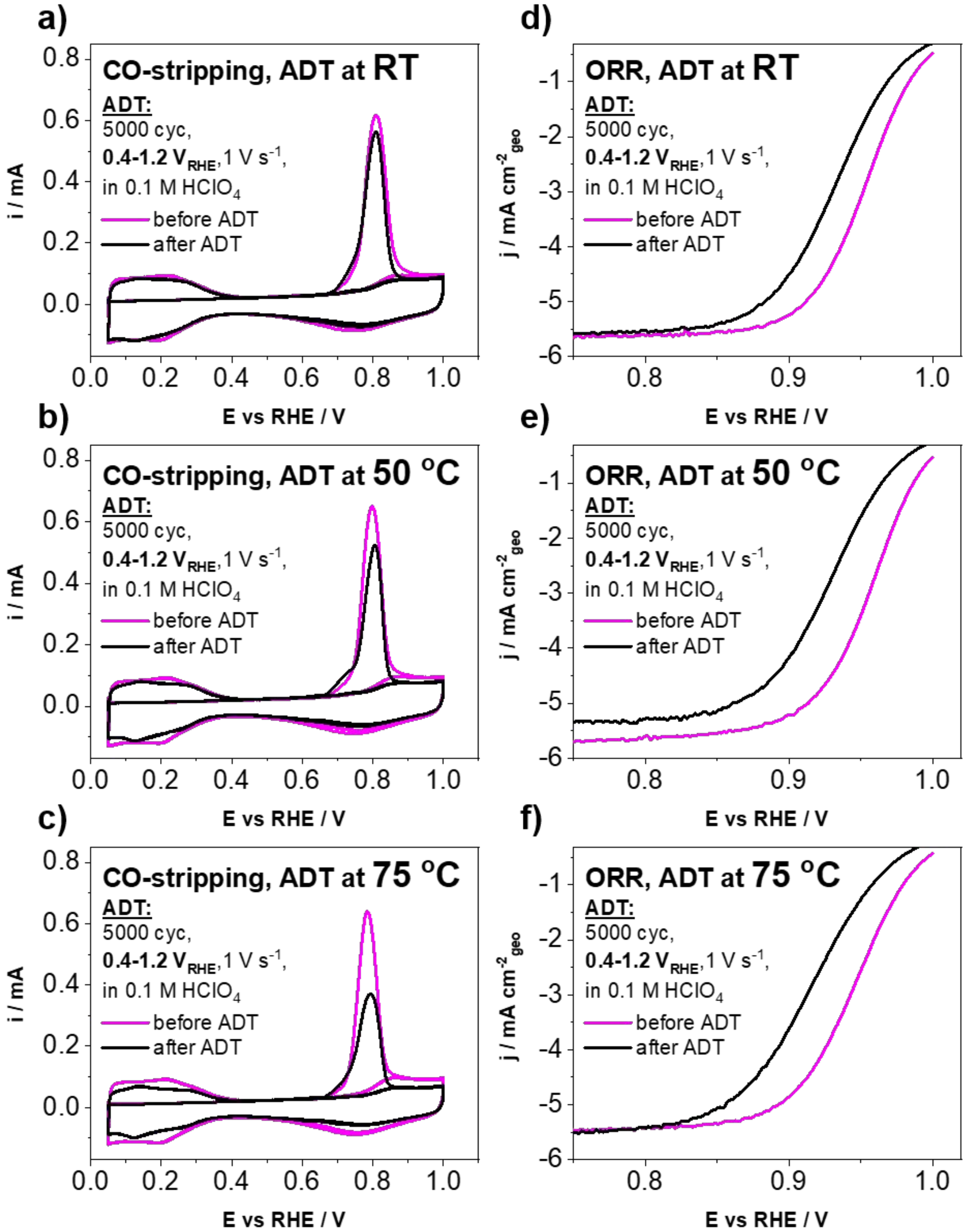

Figure S12: Comparison of **(a-c)** the CO-electrooxidation CVs as well as the follow-up cycles and **(d-f)** ORR polarization curves before and after the ADT performed at RT, 50 °C or 75 °C between 0.4-1.2 V_{RHE} (5000 cycles, 1 V s^{-1}, 0.1 M $HClO_4$) for the experimental Pt-Co ReCatalyst electrocatalyst.

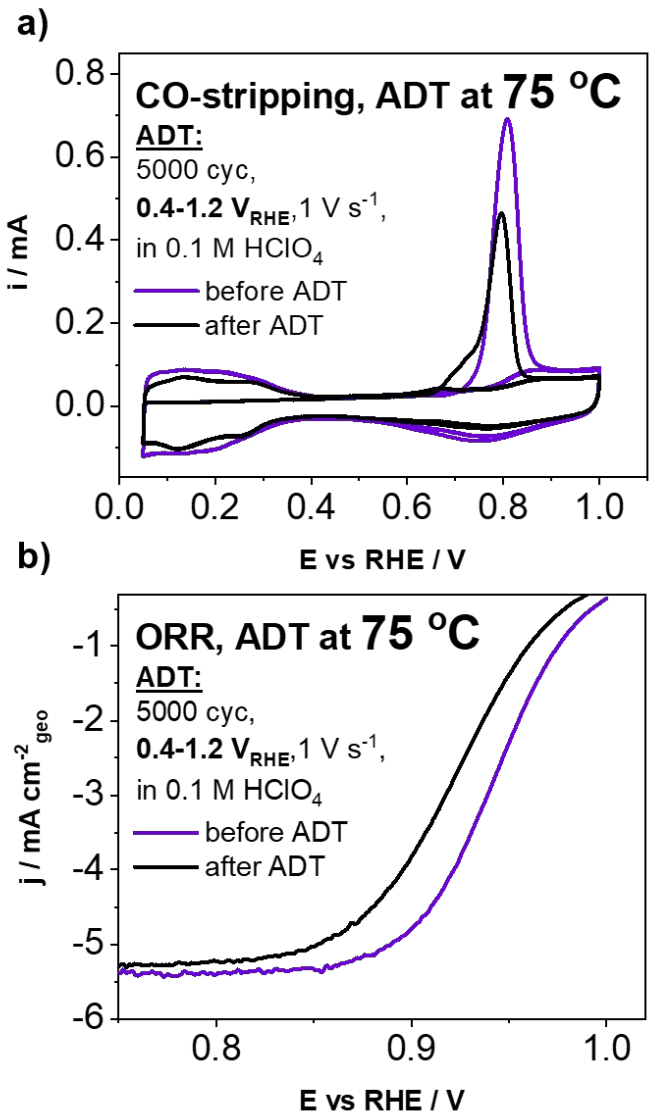

Figure S13: Comparison of **(a)** the CO-electrooxidation CVs as well as the follow-up cycles and **(b)** ORR polarization curves before and after the ADT performed at 75 °C between 0.4-1.2 V_{RHE} (5000 cycles, 1 V s^{-1}, 0.1 M $HClO_4$) for Elyst Pt50 0690 benchmark from Umicore.

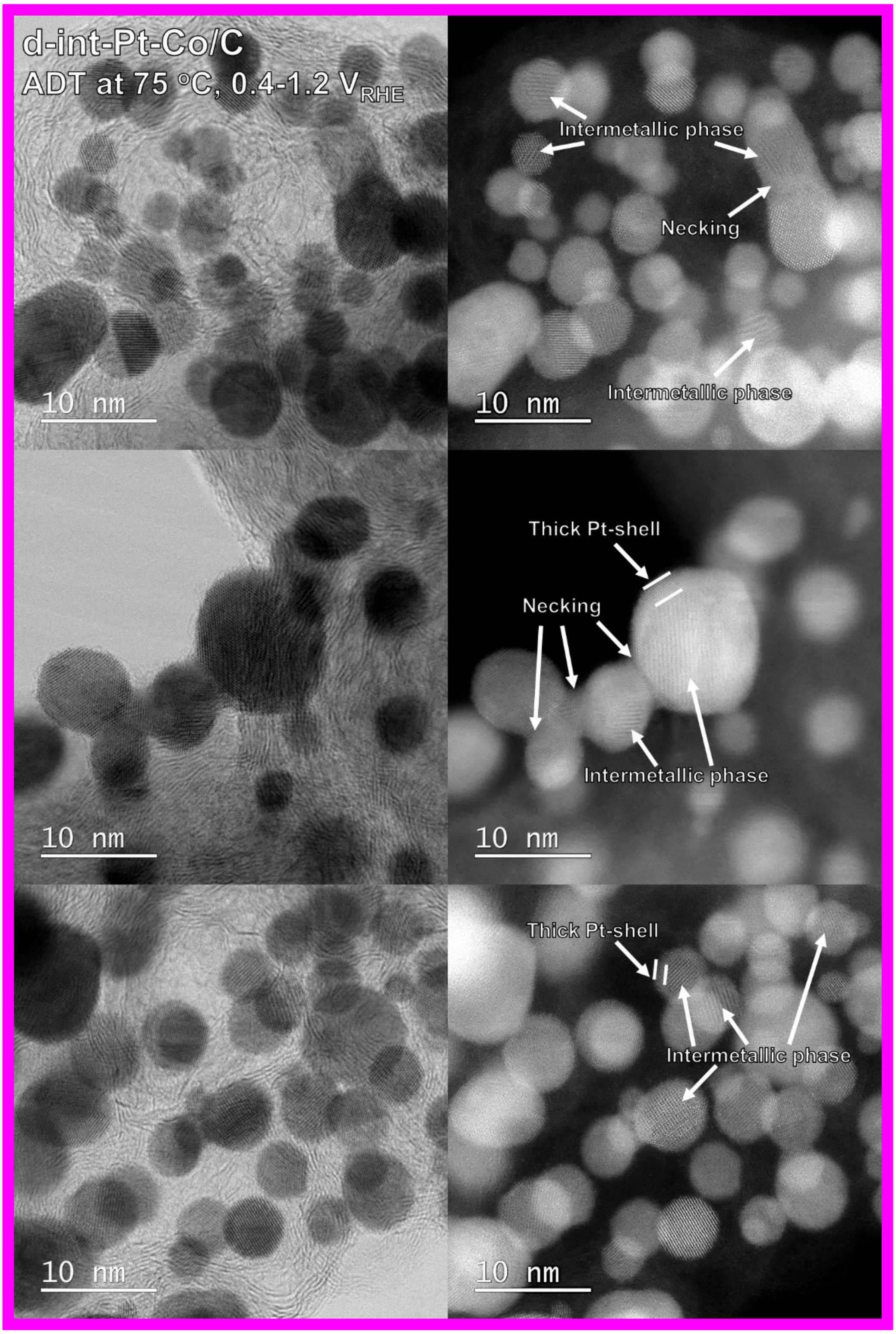

Figure S14: *Ex-situ* STEM and BF images of the experimental d-int-Pt-Co/C ReCatalyst electrocatalyst after the ADT performed at 75 °C between 0.4-1.2 V_{RHE} (5000 cycles, 1 V s^{-1}, 0.1 M $HClO_4$). In all Figures, magenta is used for the data corresponding to the experimental ReCatalyst electrocatalyst.

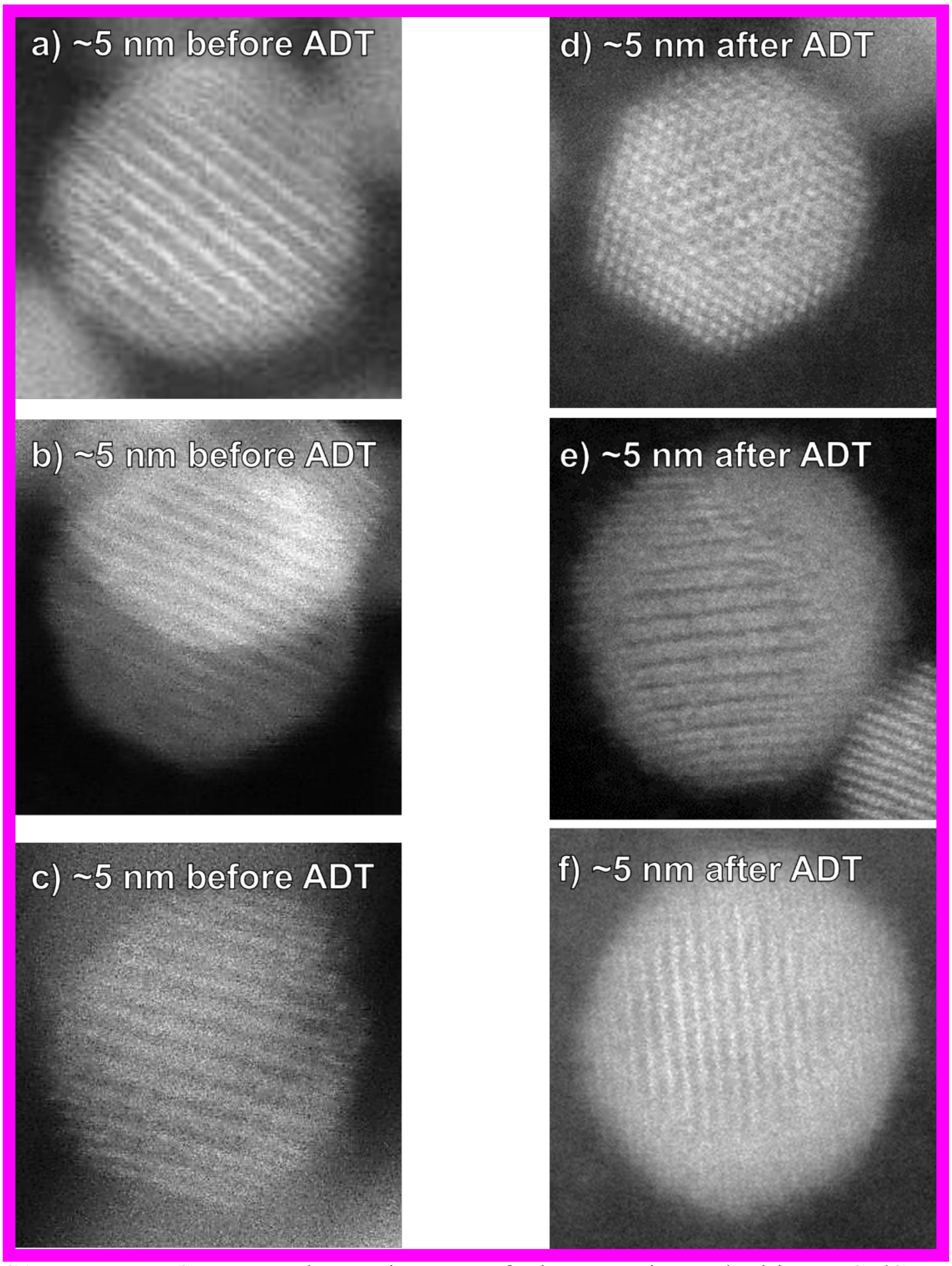

Figure S15: *Ex-situ* STEM and BF images of the experimental d-int-Pt-Co/C ReCatalyst electrocatalyst (individual representative nanoparticles) before and after the ADT performed at 75 ^{o}C between 0.4-1.2 V_{RHE} (5000 cycles, 1 V s^{-1}, 0.1 M $HClO_4$). In all Figures, magenta is used for the data corresponding to the experimental ReCatalyst electrocatalyst.

References:

(1) Maselj, N.; Gatalo, M.; Ruiz-Zepeda, F.; Kregar, A.; Jovanovič, P.; Hodnik, N.; Gaberšček, M. The Importance of Temperature and Potential Window in Stability Evaluation of Supported Pt-Based Oxygen Reduction Reaction Electrocatalysts in Thin Film Rotating Disc Electrode Setup. *J. Electrochem. Soc.* **2020**, *167* (11), 114506. https://doi.org/10.1149/1945-7111/aba4e6.
